# Physiological mechanisms contributing to the QTL *qDTY*_*3.2*_ effects on improved performance of rice Moroberekan x Swarna BC_2_F_3:4_ lines under drought

**DOI:** 10.1186/s12284-018-0234-1

**Published:** 2018-07-31

**Authors:** Alexandre Grondin, Shalabh Dixit, Rolando Torres, Challa Venkateshwarlu, Eric Rogers, Thomas Mitchell-Olds, Philip N. Benfey, Arvind Kumar, Amelia Henry

**Affiliations:** 1 International Rice Research Institute, Los Baños, Laguna Philippines; 2 International Rice Research Institute South Asia Hub, ICRISAT, Patancheru, Telangana India; 3 Department of Biology and Howard Hughes Medical Institute, Duke University, Durham, NC USA; 4 Present address: UMR DIADE, Institut de Recherche pour le Développement/Université de Montpellier, Montpellier, France

**Keywords:** Rice, Drought, Quantitative trait locus, Root system architecture, Canopy temperature, Flowering

## Abstract

**Background:**

Traditional rice (*Oryza sativa*) varieties are valuable resources for the improvement of drought resistance. *qDTY*_*3.2*_ is a drought-yield quantitative trait locus that was identified in a population derived from the traditional variety Moroberekan and the drought-susceptible variety Swarna. In this study, our aim was to characterize the physiological mechanisms associated with *qDTY*_*3.2*_. Our approach was to phenotype fifteen BC_2_F_3:4_ lines for shoot and root drought resistance-related traits as compared to Swarna in the field under well-watered and drought stress conditions. Four BC_2_F_3:4_ lines contrasting for yield under drought were selected for detailed characterization of shoot morphology, water use related traits, flowering time and root system architecture in the field as well as in controlled environments (lysimeters in a greenhouse, and gel imaging platform in a growth chamber).

**Results:**

Across five field experiments, grain yield correlated significantly with root growth along the soil profile, flowering time, and canopy temperature under drought conditions. The four selected BC_2_F_3:4_ lines showed earlier flowering time, reduced distribution of root growth to shallow soil layers which resulted in lower water uptake (between 0 and 30 cm) and drought-induced increased distribution of root growth to deep soil layers (between 30 and 60 cm) as compared to Swarna in the field. Root system architecture phenotypes were confirmed in whole root systems in lysimeters, and corresponded to higher numbers of root tips in a gel imaging platform, highlighting the potential stability of some root traits across different growth stages and systems.

**Conclusions:**

We conclude that earlier flowering time, reduced shallow root growth, and drought-induced increased deep root growth are associated with the presence of *qDTY*_*3.2*_ since these phenotypes were consistently observed in the selected QTL lines with full introgression of *qDTY*_*3.2*_. We hypothesize that the *qDTY*_*3.2*_ associated RSA phenotypes led to better use of water and metabolic resources which, combined with earlier flowering time, improved yield under drought.

**Electronic supplementary material:**

The online version of this article (10.1186/s12284-018-0234-1) contains supplementary material, which is available to authorized users.

## Background

Water limitation is responsible for major losses in rice (*Oryza sativa*) yield, and this situation is likely to worsen based on climate projections and increased competition for fresh water (Wheeler and von Braun 2013). Therefore, increasing rice yield and maintaining its stability under limited water conditions is a major challenge to improve food security. Developing drought resistant rice varieties, as in other crops, mostly relies on improving the ability to access, capture and transport water, on using this water efficiently in a compromise with carbon fixation, and on allocating the carbohydrates towards the grain (Tuberosa 2012).

Roots play a central role in maintaining the water status of the whole plant (Maurel et al. 2010). Root system architecture (RSA), which describes the spatial root arrangement within the soil, can affect the efficiency of nutrient and water extraction with further benefits to plant fitness and yield under drought (Uga et al. 2013; Lynch 2015; Lilley and Kirkegaard 2016). Proposed RSA “ideotypes” for drought resistance include traits such as deep root growth, root thickness, lateral root length and density of specific root types (Ahmadi et al. 2014; Lynch et al. 2014). Techniques involving root phenotyping in controlled conditions have led to the identification of a number of genetic determinants of RSA traits in rice (Uga et al. 2011; Topp et al. 2013; Courtois et al. 2013), but their role in controlling water extraction and plant water status remains to be validated in agronomic conditions (Langridge and Reynolds 2015). Furthermore, RSA and its effects on root hydraulics have to be integrated in a whole plant strategy, where the shoot can equally affect plant water status by regulating water use through leaf area and stomatal aperture (Vadez 2014). The complex interactions between root water extraction and shoot water use under drought stress are far from being understood but may, from a yield perspective, rely on how plants manage the decreasing water resource in a way that water is still available at reproductive stage (Lynch 2013; Vadez et al. 2014). In fact, the complexity of the plant response to drought and the variety of drought stress scenarios across rice cultivation systems has often restricted breeding strategies aiming at developing drought-yield resistant rice lines by targeting physiological traits (Leung 2008; Kumar et al. 2014).

Alternative strategies consist of seeking a drought-yield quantitative trait locus (QTL) without trade-offs on yield under optimum conditions through quantitative genetic approaches, and further characterization of the physiological drought response mechanisms of the breeding lines containing those QTLs (Swamy et al. 2013; Dixit et al. 2015b; Henry et al. 2015). This top-down approach allows the identification of drought resistance-related traits as whole plant strategies to increase productivity and may be relevant to multiple drought stress scenarios in targeted environments (Passioura 2012). For instance, when introgressed in the drought-susceptible high-yielding popular rice variety IR64, two major-effect drought-yield QTLs (*qDTY*_*2.2*_ and *qDTY*_*4.1*_) improved yield through higher root hydraulic conductivity and better transpiration and plant growth (Henry et al. 2015). Ultimately, the selective combination of QTLs showing distinct and potentially complementary drought-resistance physiological mechanisms will help future breeding programs.

A cross between Moroberekan, a drought- and rice blast-resistant tropical japonica variety but with poor yield potential, and Swarna, a popular semi-dwarf indica variety that shows high yield potential but is drought- and blast-susceptible, resulted in the identification of QTL *qDTY*_*3.2*_, a major-effect drought-yield QTL under severe drought stress conditions (Dixit et al. 2014). Further studies on this population in multiple agronomic situations identified a QTL cluster that co-located with *qDTY*_*3.2*_ (Dixit et al. 2015a), as well as QTLs for time to flowering (*HD9*), lodging-resistance traits, and drought resistance-related traits including canopy cover and canopy temperature. Positive correlations between grain yield and root mass density at depth were observed in the same population (Dixit et al. 2015a). These results precede more detailed physiological characterization of this population that could potentially unravel novel drought-resistance mechanisms, and support the identification of new drought-resistance genes present in this QTL.

In this study, we characterized the physiological drought-response mechanisms of *qDTY*_*3.2*_. We used a subset of 15 BC_2_F_3:4_ lines generated from the cross between Moroberekan and Swarna in which complete or partial segments of *qDTY*_*3.2*_ were fixed. Without any a priori considerations, our approach was to phenotype these lines for shoot and root morphological traits and agro-morphological traits as compared to Swarna in the field. We report here detailed results from a selection of four BC_2_F_3:4_ lines contrasting for yield under drought that were grown for five consecutive seasons under well-watered and drought stress conditions and used for RSA characterization in controlled environments (lysimeters filled with soil in a greenhouse, and a gel imaging platform in a growth chamber).

## Methods

### Plant material

*qDTY*_*3.2*_ is a major-effect drought-yield QTL that was identified in a rice mapping population of 361 BC_2_F_3:4_ lines developed from the cross between Moroberekan and Swarna (recurrent parent) by Dixit et al. (2014). A subset of 15 BC_2_F_3:4_ QTL lines that were fixed for *qDTY*_*3.2*_ were selected from that population based on contrasting performance under drought and used in this study (Additional file 1: Table S1). Among the 15 selected QTL lines, detailed measurements were conducted in field, lysimeter and gel imaging platform studies on 252-B (IR 91648-B-252-B) in which a partial segment of *qDTY*_*3.2*_ was present, and 73-B (IR 91648-B-73-B), 33-B (IR 91648-B-33-B), and 89-B (IR 91648-B-89-B), in which a complete segment of *qDTY*_*3.2*_ was present (Additional file 1: Table S1 and Additional file 2: Figure S1). To verify the robustness of the QTL effect, five advanced BC_2_F_3:6_ lines were further studied (Additional file 1: Table S1).

### Field experiments

Field experiments were conducted at the IRRI experimental station (14°11′N, 121° 15′E) during the dry seasons (DS) of 2013, 2014 and 2015, and during the wet seasons (WS) of 2013 and 2014. The dry seasons were generally from mid-December to mid-April, and the wet seasons were from the beginning of June to the end of October. Experiments were labeled by year and season (e.g., 13DS for dry season or 13WS for wet season of 2013) and included a well-watered and a drought stress treatment (Table 1). After observing large differences in flowering time in Experiment 13DS, the QTL lines were divided into two maturity groups (Early: E and Late: L) in Experiments 13WS and 14DS in order to apply the drought stress treatment at a similar developmental stage. Due to field space limitations, Experiments 14WS and 15DS were not separated into maturity groups.

**Table 1:** Mean trial yield of rice genotypes (Swarna, Moroberekan and QTL lines) in each field experiment of this study

Time of year	Experiment name	Initiation of drought stress treatment (DAS)	Mean trial yield (kg ha^− 1^) in well-watered treatment	Mean trial yield (kg ha^− 1^) in drought stress treatment
Dry season 2013	13DS	75	4951	211
Wet season 2013	13WS (E)	47	2567	313
	13WS (L)	75	2825	399
Dry season 2014	14DS (E)	60	3231	104
	14DS (L)	70	3754	192
Wet season 2014	14WS	51	3911	2132
Dry season 2015	15DS	70	4916	452

During the wet season 2013 and dry season 2014, QTL lines were separated into two maturity groups (E: early and L: late). Except in Experiment 14WS, rainfall was excluded from the drought stressed plots using an automated rolling rain-out shelter. DAS: days after sowing

In all field experiments, seedlings were established in a wet bed nursery for 21 days before being transplanted in puddled and bunded fields under lowland conditions. Fields were divided into 2.1-3 m^2^ plots consisting of 3 or 4 rows spaced at 0.25 m, each row containing 15 hills spaced at 0.20 m. The experiments were laid out in a randomized complete block design with four replications. Plots in the well-watered treatment were maintained flooded. The drought stress plots were established in a rain-out shelter facility about 15 m away from the well-watered plots. Depending on the season and the maturity group, plots in the drought stress treatment were maintained flooded from transplanting until 47 to 75 days after sowing (DAS), after which drought was initiated by withholding irrigation and rainfall exclusion using an automated rainout shelter. In Experiment 14WS, the same protocol of drought imposition was applied except that the rainout shelter was used only from 51 to 68 DAS; 432 mm of rainfall occurred from 68 DAS to maturity resulting in a moderate drought stress treatment in 14WS. In the drought stress treatment, soil moisture at a depth of 30 cm was monitored using tensiometers (Soilmoisture Equipment Corp., CA, USA). Drought treatments were re-irrigated when the soil moisture dropped below -60 kPa at 30 cm depth; therefore each genotype experienced some degree of progressive drydown and recovery in all trials, regardless of maturity grouping. Complete fertilizer was applied before transplanting at the rate of 40–40-40 kg ha^− 1^ NPK. At about panicle initiation stage, ammonium sulfate was top-dressed at the rate of 40 kg N ha^− 1^. All fields were maintained free of weeds.

### Phenology, grain yield and morphology in the field

The number of days from sowing to flowering (time to flowering; DTF) was recorded when 50% of the plants in a plot had flowered. Total leaf area and average leaf width were measured on three plants per plot using a roller-belt-type leaf area meter (LI-3100C, LiCor, NE, USA). At maturity, plant height and tiller number were measured on three randomly sampled plants, and entire shoots were harvested from an area of 1.5 m^2^ in each plot and dried. Panicles were separated from their tillers to measure grain yield (GY) normalized to 14% grain moisture content, and leaves and tillers were weighed to determine shoot dry mass (SDM). Harvest index (HI) was calculated as the ratio of seed weight and total aboveground biomass (GY + SDM). Percent reduction in SDM and HI under drought stress were calculated in each experiment as compared to the averaged value of SDM and HI observed for each genotype in the well-watered treatment.

Root sampling was performed at maturity in the well-watered treatment and after re-watering the soil in the drought stress treatment using a 4-cm-diameter steel tube by soil coring to a depth of 60 cm as described by Henry et al. (2015). The soil core was divided into four segments of 15 cm length and roots of each segment were carefully washed. Roots were then scanned at 600 dpi (Epson V700, CA, USA) and analyzed for total root length using WinRhizo (Regent Instruments, Quebec, Canada). Root length density (RLD) was calculated as the total root length divided by soil volume. The percentage of shallow and deep roots was calculated from the total root length measured from 0 to 30 cm and 30–60 cm soils, respectively, and divided by the total root length measured from the entire soil core (0–60 cm) × 100. Crown root number, referring to the number of roots emerging from the root-shoot junction, was counted after plant excavation to a depth of approximately 10 cm.

To validate the trends observed to be related to *qDTY*_*3.2*_ in comparison with multiple lines that were negative for the QTL, we re-analyzed root mass data reported by the QTL co-location study of Dixit et al. (2015a). A total of 85 BC_2_F_3:4_ lines from that study grown in an early maturity group with or without the full QTL region were classified as “−*qDTY*_*3.2*_” (18 lines) or “+ *qDTY*_*3.2*_” (37 lines) based on four SNP markers spanning the QTL region. Lines with partial introgressions of the QTL region were excluded from the analysis. The percentage of shallow and percentage of deep roots were calculated from the root dry mass (RDM) from the shallow soil layer (0–15 cm) or the deep soil layer (45–60 cm) divided by the root mass acquired from the entire soil core (0–60 cm) × 100.

### Water use related traits measurements in the field

In the field experiments, canopy temperature (CT) and within-plot volumetric soil moisture (a proxy for plant water uptake) were conducted in the drought stress treatments only. Among them, canopy temperature was measured on sunny days typically between 1100 h and 1200 h at multiple locations per plot using infrared sensors (Apogee Instruments, UT, USA) mounted on a semi-automated sensor rack. Volumetric soil moisture was monitored at three locations per plot by frequency domain reflectometry through PVC tubes (Diviner 2000, Sentek Sensor Technologies, SA, Australia) allowing estimation of root water uptake at different soil depths after a re-watering event. Chlorophyll fluorescence, carbon isotope discrimination and stomatal density were also measured following protocols described in the legends of Additional file 3: Figure S2, Additional file 4: Figure S3 and Additional file 5: Figure S4, respectively.

### Water uptake and root morphology in lysimeters

The lysimeter experiment was conducted in a greenhouse at IRRI from mid-December 2013 to mid-February 2014 as described by Kijoji et al. (2012). The lysimeters (PVC cylinders of 95 cm height and 20 cm diameter) were arranged in a randomized complete block design with five replications. Seeds were germinated in petri-dishes for 4 days before transplanting into lysimeters containing basal complete fertilizer at a rate of 0.3 g kg^− 1^. The soil was kept saturated until 31 DAS to allow plant establishment. The drought treatment was initiated at 32 DAS by withholding water and opening the drainage holes located at the bottom of the lysimeters. Lysimeters were weighed three times per week until 63 DAS in order to determine the amount of water needed to keep the soil saturated in the well-watered treatment (corresponding to their weight at 31 DAS) and to monitor the dry-down in the drought stress treatment. No water was added to the drought-treated lysimeters from the start of the dry-down to harvest.

Total water uptake (TWU) was calculated as the cumulative water loss by the drought-treated lysimeters from 32 to 63 DAS. Roots from the 0–20, 20–40 and 40–60 cm soil segments were washed, oven dried and the percentage of shallow (0–20 cm) and percentage of deep (20–60 cm) roots were calculated as described above.

### Root system architecture assayed with a gel imaging platform

Selected QTL lines were grown, imaged, and phenotyped for RSA using the semi-automated root imaging and analysis pipeline developed at Duke University (Durham, NC, USA) according to Topp et al. (2013). Briefly, dehulled and sterilized seeds were germinated in petri dishes containing Yoshida’s nutrient solution solidified using 0.2% Gelzan gellan gum (Caisson Laboratory, USA) for three days in the dark at 28 °C. Seedlings were then transferred into cylinders (82.5 mm diameter × 520 mm high) filled with the nutrient/Gelzan solution alone (control; C) or complemented with 10% polyethylene glycol 8000 (PEG; Sigma Aldrich, USA) resulting in an osmotic pressure of − 0.25 MPa at 25 °C and simulating a water deficit treatment (WD). Seedlings were grown for 12 days in a growth chamber (12 h day/night, 28 °C day and 25 °C night) and root systems were imaged in a 360° view using a computer-controlled camera at 15 DAS (seedling stage). Images were further processed using GiA Roots (www.giaroots.org) in order to obtain 2D RSA trait measurements. Roots were analyzed for surface area (SA), total root length (TL), depth, width, and maximum number of roots (MNR).

### Statistics

Statistical analyses were performed with R v. 3.2.2 (R core team 2017). Correlation analyses between GY and other measured traits were performed within each experiment using the Pearson’s correlation test of the rcorr function. ANOVA (aov function) was performed across experiments or dates in order to detect significant differences between genotypes (G), experiments (E) and genotype x experiment (G x E). Tukey’s Honest Significant Differences (HSD function) post-hoc test was used to group genotypes into letter classes.

## Results

### Correlation between grain yield, shoot and root growth-related traits in the QTL lines

In order to confirm the effect of *qDTY*_*3.2*_ on grain yield and identify the physiological mechanisms associated with this QTL, 15 BC_2_F_3:4_ lines were grown in the field along with Swarna and Moroberekan under well-watered and drought stress conditions in Experiment 13DS and Experiments 13WS and 14DS in which genotypes were divided into two maturity groups. These 15 lines were all positive for the presence of *qDTY*_*3.2*_ (with lines 177-B, 192-B and 252-B showing partial introgression; Additional file 1: Table S1) but were contrasting for grain yield (GY) under drought stress as compared to Swarna (Additional file 1: Tables S2 and S3). In Experiments 14WS and 15DS, a selection of BC_2_F_3:4_ lines contrasting for their yield responses under drought were grown along with a selection of BC_2_F_3:6_ advanced generation lines (Additional file 1: Table S1). As an indication of the drought stress intensity, mean trial yields were calculated in each experiment, with low mean trial yield corresponding to severe drought stress conditions (< 452 kg ha^− 1^ in Experiments 13DS, 13WS, 14DS and 15DS) and high mean trial yield (> 3000 kg ha^− 1^) corresponding to favorable (well-watered) conditions (Table 1). In Experiment 14WS the drought stress was moderate with a mean trial yield of 2132 kg ha^− 1^.

To have a broader view of the relationship between GY and drought resistance-related traits, we performed correlation analyses between GY and tiller number (Tiller), canopy temperature (CT), time to flowering (DTF), shoot dry mass (SDM), root length density (RLD) and % shallow and deep roots which were consistently measured on the all lines grown in each field experiment under well-watered and drought stress conditions (Table 2). Under well-watered conditions, GY was significantly positively correlated with SDM in Experiments 13DS, 14DS (E) and 14WS, and with RLD at 0–30 cm in Experiments 13WS (L), 14DS (E) and 14DS (L). Under drought stress, GY was significantly negatively correlated with DTF in Experiments 13DS and 13WS, and with CT in Experiments 13DS and 15DS. When considering the most severe drought experiments (i.e., all except 14WS), GY was generally negatively correlated with % shallow roots (significant in Experiments 13DS and 14DS). Conversely, GY was positively and significantly correlated with RLD at 30–60 cm and % deep roots in those experiments. Overall, although correlation coefficients were low in most cases (< 0.5), *p*-values suggested that improved GY under severe drought conditions in that panel of *qDTY*_*3.2*_ lines was most consistently associated with reduction in canopy temperature, earlier flowering time, reduction in shallow root growth and increase deeper root growth.

**Table 2:** Correlation of shoot- and root-growth related traits with grain yield across field experiments under well-watered and drought stress conditions

Treatment	Trait	Correlation with grain yield						
		13DS	13WS (E)	13WS (L)	14DS (E)	14DS (L)	14WS	15DS
Well-watered	Tiller	0.1450	−0.0776	0.2274	0.2458	0.3289*	0.1768	0.1157
	DTF	−0.0688	−0.2478	−0.1718	0.1065	0.1072	0.166	0.1315
	SDM	0.631***	0.204	0.2097	0.4034*	0.0418	0.3628*	n.a.
	RLD (0–30)	n.a.	−0.1263	0.3543*	0.4301**	0.4663**	0.2656	0.1914
	RLD (30–60)	n.a.	−0.1396	0.0758	0.1021	0.1387	0.0851	−0.1768
	% shallow roots	n.a.	0.059	0.0236	0.1857	0.1313	0.147	0.3629
	% deep roots	n.a.	−0.059	−0.0236	−0.1857	−0.1313	−0.147	−0.3629
Drought stress	Tiller	0.0375***	−0.1439	−0.366*	−0.6481***	−0.7027***	0.1083	−0.0395
	CT	−0.4981***	−0.0487	−0.0184	−0.2379	−0.3107	0.0971	−0.4983*
	DTF	−0.5695*	−0.5117**	−0.4434**	0.247	−0.2042	−0.244	−0.2469
	SDM	0.2439**	−0.203	0.1347	−0.1086	0.1957	0.1326	n.a.
	RLD (0–30)	−0.3528*	0.2557	0.0307	−0.0156	−0.5285***	0.4113*	−0.2872
	RLD (30–60)	0.3048*	0.1509	0.1267	0.6523***	0.4814**	−0.1126	−0.1282
	% shallow roots	−0.5077***	0.0523	−0.1381	−0.438**	−0.7437***	0.4684**	−0.0896
	% deep roots	0.5077***	−0.0523	0.1381	0.438**	0.7437***	−0.4684**	0.0896

Traits measured on the BC_2_F_3:4_ lines included the well-watered and drought stress treatments of Experiments 13DS, 13WS (E), 13WS (L), 14DS (E), 14DS (L), 14WS and 15DS were used for Pearson correlation test. Tiller: number of tiller; CT: canopy temperature; DTF: time to flowering; SDM: shoot dry mass; RLD (0–30): root length density from 0 to 30 cm; RLD (30–60): root length density from 30 to 60 cm; % shallow roots: percentage of root length from 0 to 30 cm; % deep roots: percentage of root length from 30 to 60 cm. Values indicate the Pearson’s coefficient of correlation. *p-values < 0.05; **p-values < 0.01; ***p-values < 0.001. n.a.: not applicable because the trait was not measured in the experiment

### Selection of four *qDTY*_*3.2*_ lines with contrasting yield under drought

**Fig. 1:**
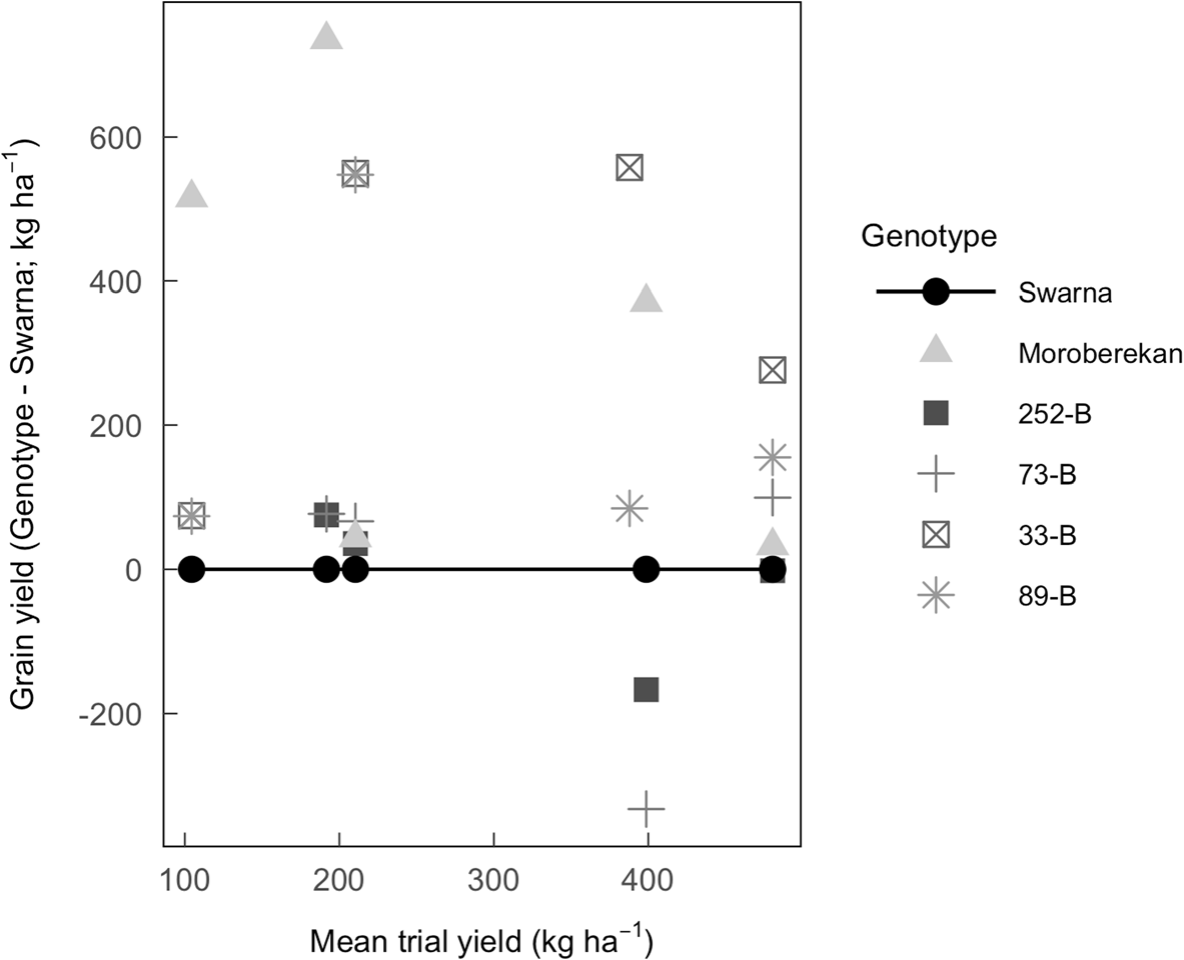
Difference in grain yield between the four selected QTL lines and Swarna under severe drought stress in the field. Each point represents the mean of the difference in grain yield (*n* = 4) between one genotype and Swarna in the drought stress treatment of Experiments 13DS, 13WS, 14DS and 15DS expressed as mean trial yield (Table 1). Actual grain yield values are presented in Additional file 2: Table S2 and Additional file 3: and Figure S3

Among traits correlated with increased grain yield, we aimed to differentiate traits associated with the presence of *qDTY*_*3.2*_ from those potentially associated with additional chromosomal introgression from the Moroberekan background. For this, we selected four lines (252-B, 73-B, 33-B and 89-B) among the subset of 15 BC_2_F_3:4_ lines that were contrasting for yield under drought. These four lines were used for further physiological characterization in field Experiments 14WS and 15DS and detailed RSA characterization in the lysimeters and gel imaging platform. 33-B and 89-B consistently showed a yield advantage compared to Swarna under severe drought stress (Fig. 1). Conversely, 252-B and 73-B showed a yield penalty or low yield advantage over Swarna under severe drought stress (Fig. 1). However, under moderate drought stress conditions 73-B showed similar yield advantage to 33-B and 89-B as compared to Swarna (Additional file 1: Table S3). Therefore, these four lines were contrasting for yield advantage under drought as compared to Swarna, considering 33-B and 89-B as the most drought resistant lines, 73-B as a moderate drought resistant line and 252-B as the least drought-resistant line. Of these genotypes, full introgressions of *qDTY*_*3.2*_ were present in 33-B, 89-B, and 73-B, and a partial introgression of *qDTY*_*3.2*_ was present in 252-B (Additional file 1: Table S1).

### Shoot morphology and water use related traits in field conditions

**Fig. 2:**
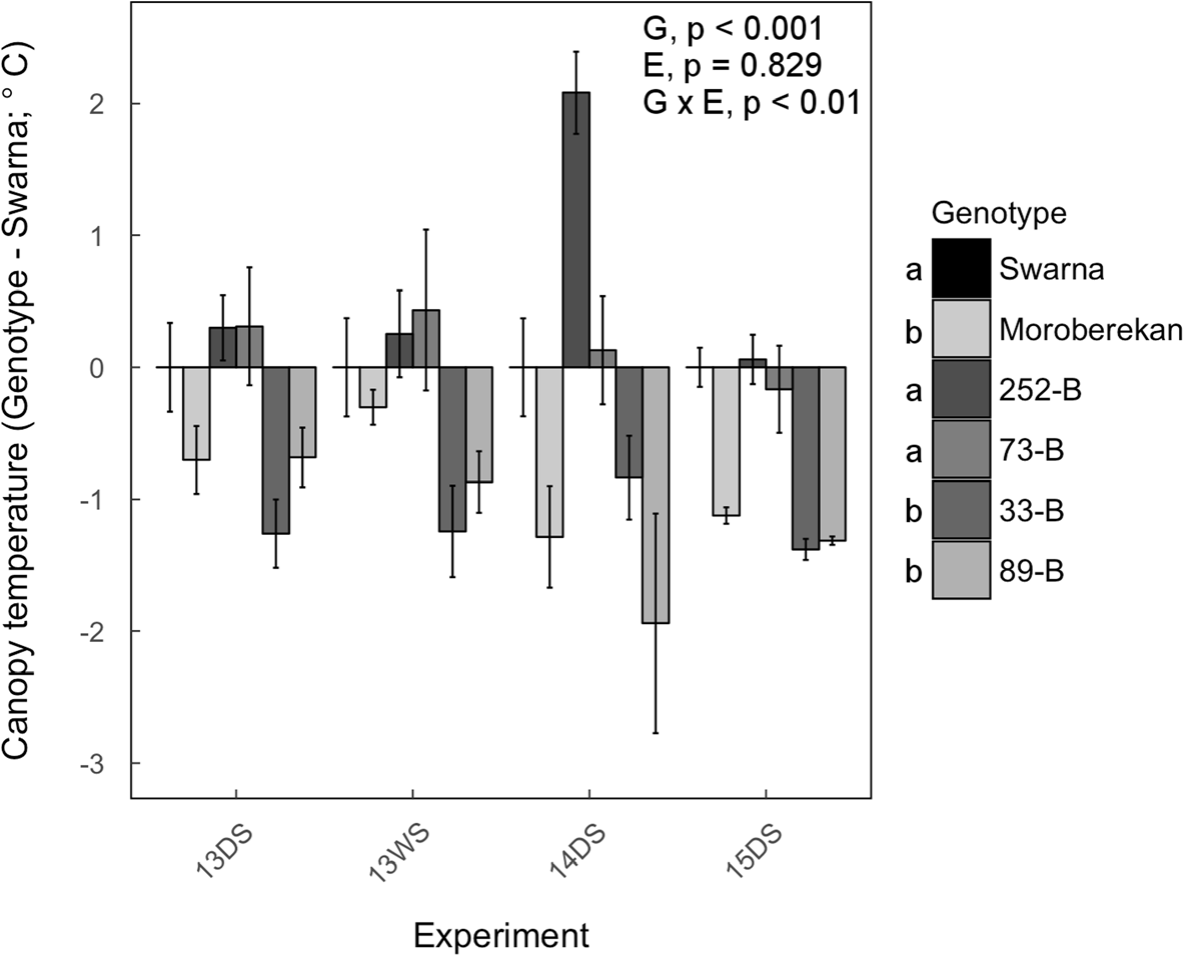
Difference in canopy temperature between the four selected QTL lines and Swarna under drought stress in the field. Bars represent the mean of the difference in canopy temperature ± se (*n* = 4) between one genotype and Swarna in a particular drought stress experiment. Actual values of canopy temperature are presented in Additional file 10: Figure S9. *P*-values shown are for genotypic (G), experimental (E) and genotype × experiment (G × E) effects across experiments and letters indicate different significance groups

To assess if SDM as well as other shoot morphological traits and canopy temperature were associated with the presence of *qDTY*_*3.2*_, we evaluated if the differences observed in those traits between the QTL lines and Swarna were consistent in field experiments. Under both well-watered and drought stress conditions, the four selected QTL lines typically showed variable tiller numbers, total leaf area and average leaf width, with values intermediate to those of Swarna and Moroberekan (Additional file 1: Tables S4, S5 and S6). The plant height of 252-B and 73-B was similar to Swarna while the plant height of 33-B and 89-B was similar to Moroberekan (i.e., taller than Swarna), particularly under drought stress (Additional file 1: Table S7). Severe drought stress in Experiments 13DS, 13WS and 14DS induced reduction in SDM (26 to 67%) and severe reduction in HI (37 to more than 90%), but no consistent differences were observed among genotypes (Tables 3 and 4, and Additional file 1: Tables S8 and S9). Canopy temperature of 252-B and 73-B was generally higher than in Swarna across the period of the drought stress in all experiments (Fig. 2 and Additional file 3: Figure S2). In contrast, 33-B, 89-B, and Moroberekan showed slower increase in canopy temperature than Swarna under drought stress (Additional file 3: Figure S2) resulting in significantly cooler average canopy temperature (around − 1 °C) across all drought stress periods of all experiments (Fig. 2). Additional physiological traits (chlorophyll fluorescence, carbon isotope discrimination and stomatal density) were also assessed but did not show conclusive genotypic differences (Additional file 4: Figure S3, Additional file 5: Figure S4 and Additional file 6: Figure S5). Overall, differences in shoot morphology, HI and canopy temperature were variable among lines and therefore not specifically associated with the presence of *qDTY*_*3.2*_.

**Table 3:** Percent reduction in shoot dry mass under drought stress in field conditions

Genotype	Experiment					
	13DS	13WS (E)	13WS (L)	14DS (E)	14DS (L)	14WS
Swarna	67 ± 1 a	28 ± 8	37 ± 2	58 ± 2 a	46 ± 5	−9 ± 4
Moroberekan	57 ± 4 bc	n.a.	32 ± 12	44 ± 3 b	42 ± 4	−6 ± 7
252-B	65 ± 1 ab	n.a.	27 ± 6	n.a.	42 ± 6	2 ± 2
73-B	67 ± 2 a	n.a.	33 ± 3	n.a.	38 ± 4	−13 ± 5
33-B	56 ± 3 c	31 ± 5	n.a.	26 ± 5 c	n.a.	5 ± 4
89-B	63 ± 2 abc	24 ± 2	n.a.	33 ± 4 bc	n.a.	−14 ± 9
Genotype (*p*-value)	< 0.05	0.177	0.13	< 0.001	0.829	0.232

In Experiments 13WS and 14 DS QTL lines were separated into two maturity groups (E: early and L: late). SDM: shoot dry mass. Mean values ± se (*n* = 3–4) are presented. Letters indicate significant difference groups within each experiment. n.a.: not applicable because the genotype was not included in the experiment. Actual values of SDM (g m^−2^) are presented in Additional file 1: Table S4

**Table 4:** Reduction in HI (%) under drought stress in field conditions

Genotype	Experiment					
	13DS	13WS (E)	13WS (L)	14DS (E)	14DS (L)	14WS
Swarna	96 ± 2 a	87 ± 4	64 ± 9 b	100 ± 0 a	99 ± 0 a	50 ± 5
Moroberekan	90 ± 4 a	n.a.	37 ± 7 c	49 ± 5 b	42 ± 9 b	44 ± 5
252-B	93 ± 2 a	n.a.	79 ± 3 ab	n.a.	95 ± 2 a	41 ± 3
73-B	91 ± 5 a	n.a.	93 ± 2 a	n.a.	95 ± 1 a	37 ± 5
33-B	63 ± 6 b	55 ± 13	n.a.	94 ± 1 a	n.a.	24 ± 5
89-B	64 ± 13 b	87 ± 4	n.a.	94 ± 1 a	n.a.	45 ± 8
Genotype (*p*-value)	< 0.05	0.053	< 0.001	< 0.001	< 0.001	0.109

In Experiments 13WS and 14 DS QTL lines were separated into two maturity groups (E: early and L: late). HI: harvest index. Mean values ± se (*n* = 3–4) are presented. Letters indicate significant difference groups within each experiment. n.a.: not applicable because the genotype was not included in the experiment. Actual values of HI are presented in Additional file 1: Table S9

### Flowering time in field conditions

**Fig. 3:**
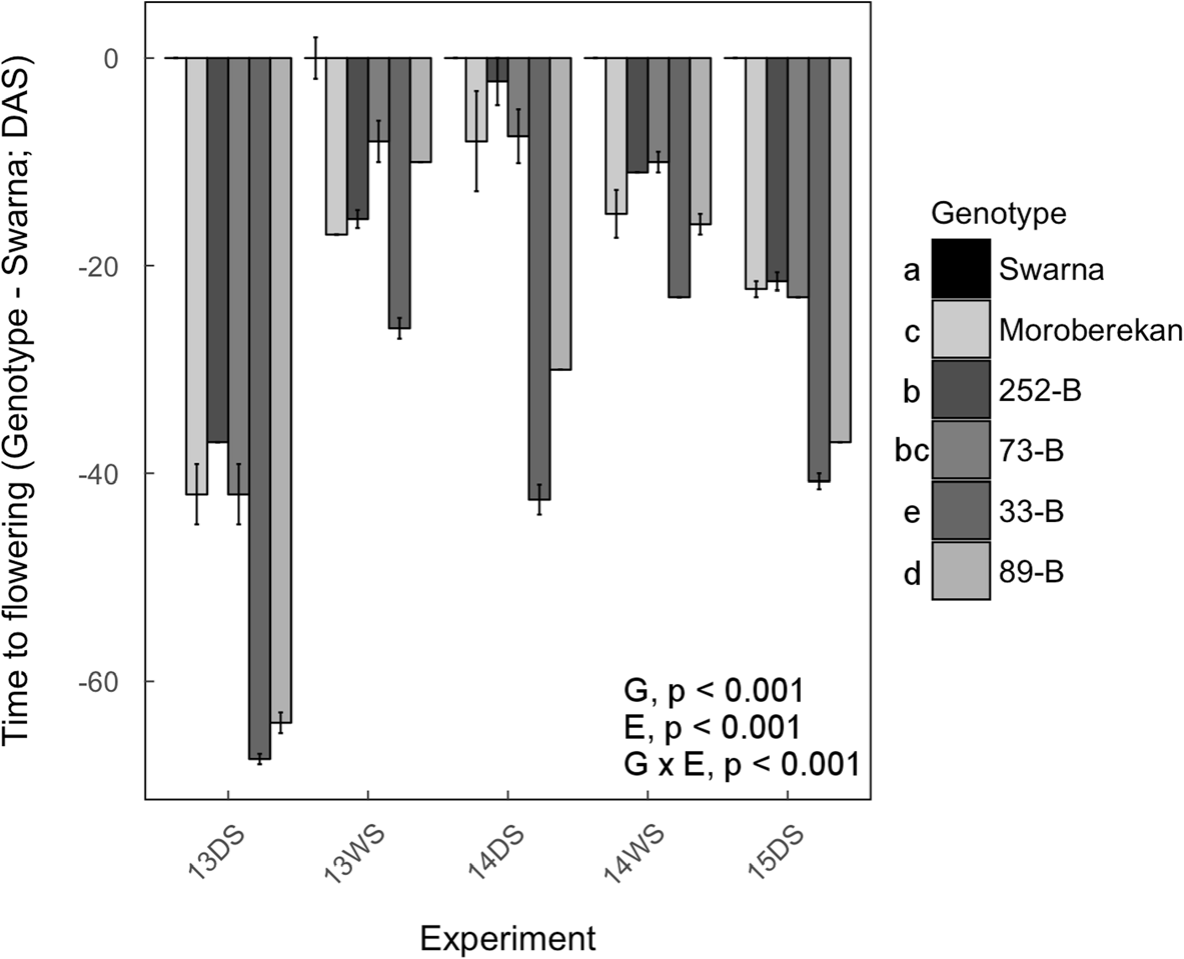
Difference in time to flowering between the four selected QTL lines and Swarna under drought stress in the field. Bars represent the mean of the difference in time to flowering ± se (*n* = 4) between one genotype and Swarna in a particular drought stress experiment. Actual values of time to flowering are presented in Additional file 7: Table S7. *P*-values shown are for genotypic (G), experimental (E) and genotype × experiment (G × E) effects across experiments and letters indicate different significance groups

To evaluate if time to flowering was associated with the presence of *qDTY*_*3.2*_, we evaluated the differences in this trait between the QTL lines, Moroberekan and Swarna. Under well-watered conditions, Swarna flowered around 100 DAS (Additional file 1: Table S10). In the same conditions, Moroberekan, 252-B, and 73-B generally flowered around 90 DAS while 33-B and 89-B flowered around 80–85 DAS. Under drought stress, flowering time was delayed from 10 to 40 days in Swarna, and was generally not affected in Moroberekan and the QTL lines (Additional file 1: Table S10). Consequently, Moroberekan, 252-B, and 73-B flowered around 20 days earlier than Swarna while 33-B and 89-B flowered around 40 days earlier than Swarna under drought stress (Fig. 3). Overall, earlier time to flowering was consistently observed (although to different degrees) in the four selected QTL lines independently of their yield performance, suggesting that this trait was associated with the presence of *qDTY*_*3.2*_.

### Root morphology in field conditions

**Fig. 4:**
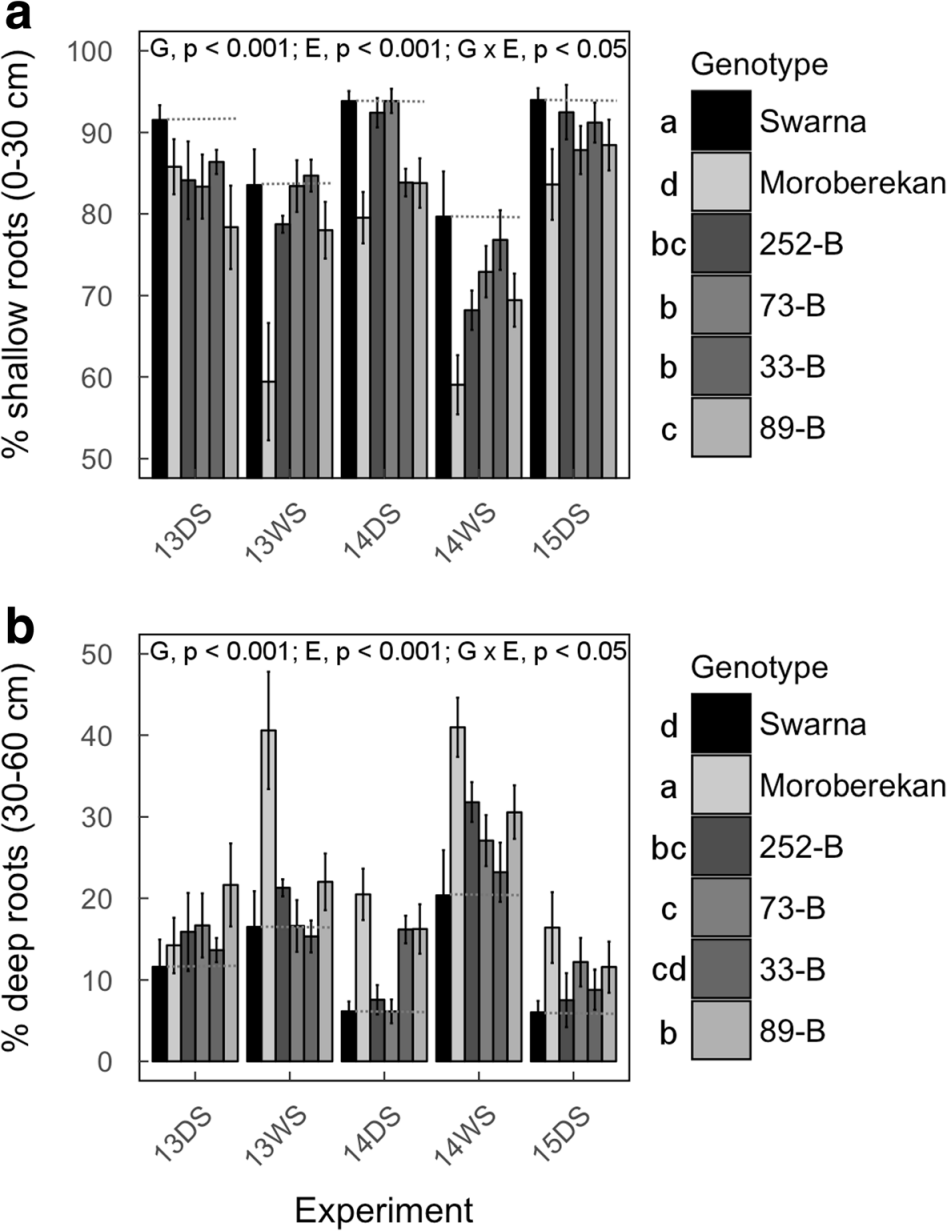
Percentage of shallow and deep root length in Swarna, Moroberekan and the four selected QTL lines under drought stress in the field. Total root length measured in 15-cm soil segments from 0 to 60 cm were analyzed to calculate the percentage (%) shallow (0–30 cm; **a**) and deep (30–60 cm; **b**) roots. Results from the late maturity group of Experiments 13WS and 14DS are presented for Swarna and Moroberekan. Bars represent mean values ± se (*n* = 3–4). *P*-values shown are for genotypic (G), experimental (E) and genotype × experiment (G × E) differences calculated across the different experiments and letters indicate different significance groups. The gray lines represent the mean value of Swarna

To investigate if the presence of the *qDTY*_*3.2*_ was associated with deeper root growth as suggested by correlation between GY and root growth profiles, we compared root morphology and architecture across genotypes in field experiments. Crown root number measured in Experiments 13DS and 14DS at maturity was generally lower in Moroberekan and the four selected QTL lines as compared to Swarna under drought stress, with significant differences observed between genotypes in 14DS (E) (*p* <  0.05) and 14DS (L) (*p* <  0.01; Additional file 1: Table S11). The percentage of shallow roots (0–30 cm) was reduced in Moroberekan and the QTL lines as compared to Swarna under drought stress in most experiments and that reduction was observed to be significant when considered across the five experiments (Fig. 4a). Similar differences among genotypes were observed for RLD measured at 0–30 cm (Additional file 7: Figure S6A). Conversely, increased percentage of deep roots (30–60 cm) was observed in Moroberekan and the QTL lines as compared to Swarna under drought stress in most experiments and that increase was significant when considered across the five experiments (Fig. 4b). Increase in RLD between 30 and 60 cm in Moroberekan and the QTL lines as compared to Swarna was observed in some experiments but no significant differences were observed across the five experiments (Additional file 7: Figure S6B). Under well-watered conditions, significant reduction in RLD was also observed in Moroberekan and the QTL lines as compared to Swarna between 0 and 30 cm while no differences between genotypes were observed in RLD below 30 cm and in percentage shallow and deep roots (Additional file 8: Figure S7 and Additional file 9: Figure S8).

**Fig. 5:**
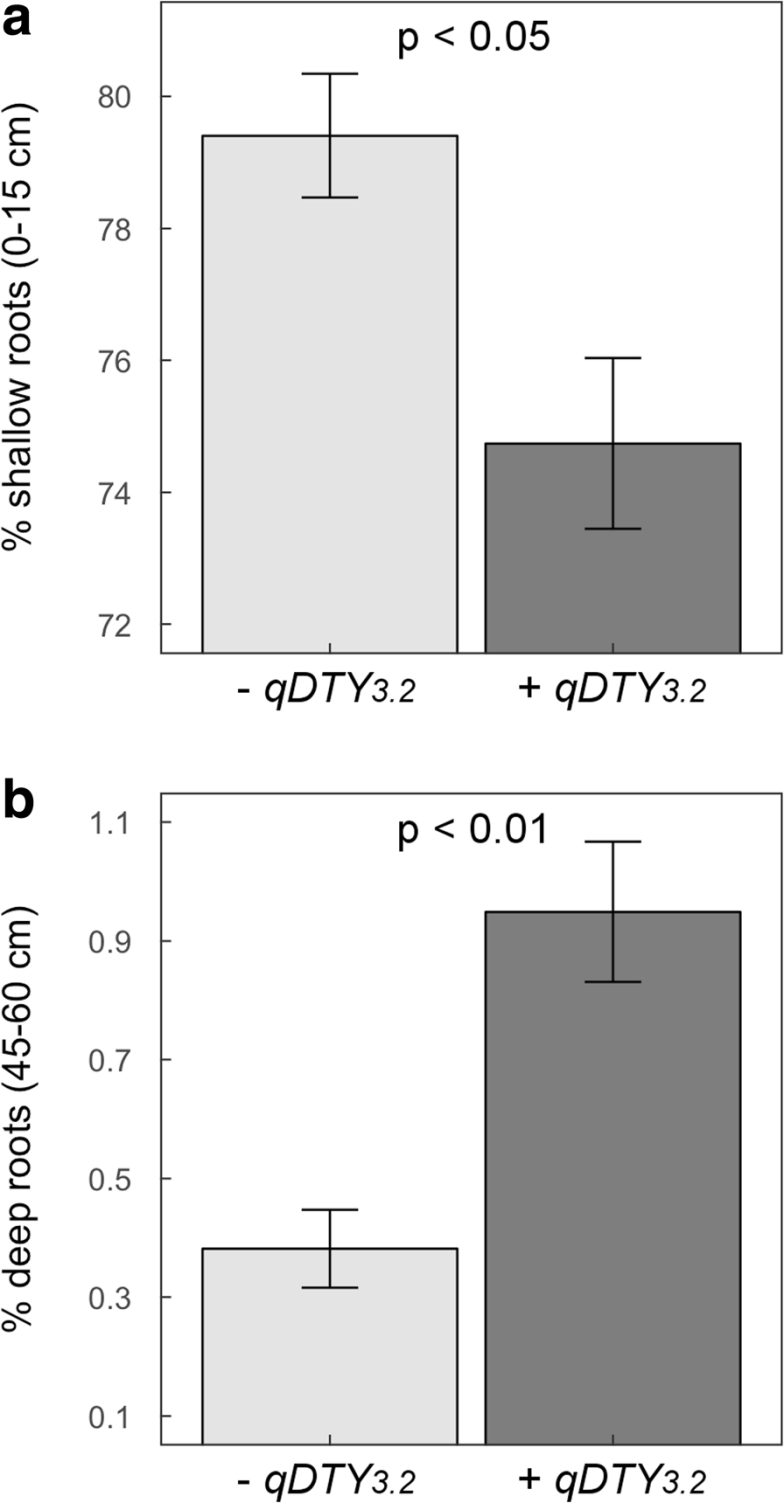
Percentage of shallow and deep root mass in QTL lines contrasting for the presence of *qDTY*_*3.2*_ in the field. Root mass density data measured in 15-cm soil segments from 0 to 60 cm and reported by Dixit et al. (2015a) were re-analyzed to calculate the percentage of shallow (0–15 cm; **a**) and deep (45–60 cm; **b**) roots in 37 lines with the *qDTY*_*3.2*_ introgression (+ *qDTY*_*3.2*_) and 18 lines without the *qDTY*_*3.2*_ introgression (− *qDTY*_*3.2*_). Bars indicate mean values ± se and *p*-values indicate differences between QTL groups

To further validate this phenotype, we performed a re-analysis of root dry mass (RDM) density from 55 QTL lines with (37 lines) or without (18 lines) the entire segment of *qDTY*_*3.2*_ introgression reported by Dixit et al. (2015a). Lines positive for the presence of *qDTY*_*3.2*_ showed significantly lower percentage shallow roots (*p* <  0.05) and showed significantly higher percentage deep roots (*p* <  0.01) as compared to lines negative for the presence of *qDTY*_*3.2*_ (Fig. 5). Overall, decreased root growth near the soil surface and increased root growth at depth were consistently observed under drought stress in both small and large panels of QTL lines in which *qDTY*_*3.2*_ was fixed, suggesting that these RSA traits were associated with the presence of *qDTY*_*3.2*_.

### Root morphology in lysimeters

**Fig. 6:**
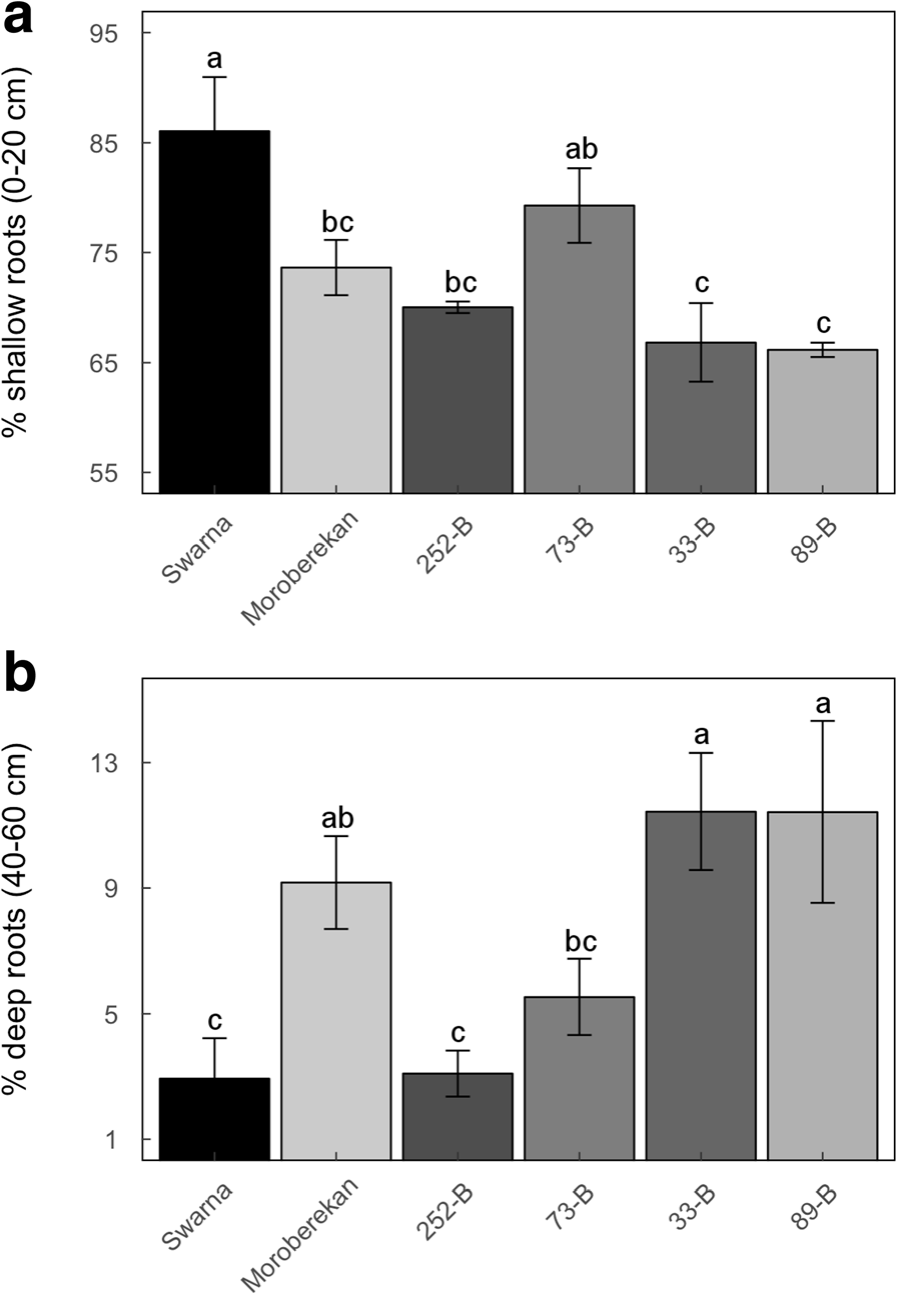
Percentage of shallow and deep root mass in Swarna, Moroberekan and the four selected QTL lines in lysimeters. Root mass density measured in 20-cm soil segments from 0 to 60 cm were analyzed to calculate the percentage (%) of shallow (0–20 cm; **a**) and deep (40–60 cm; **b**) roots. Bars indicate mean values ± se (*n* = 3) and letters indicate difference significance groups

To broaden the evaluation of root morphology, we measured RDM with depth (0–20, 20–40 and 40–60 cm) in Swarna, Moroberekan, and the four selected QTL lines grown in lysimeters. The percentage of shallow roots (0–20 cm) was significantly lower in the QTL lines and Moroberekan as compared to Swarna under drought stress (*p* <  0.01 except for 73-B; Fig. 6a). Furthermore, the percentage of deep roots (40–60 cm) in 73-B, 33-B, 89-B and Moroberekan was higher than in Swarna under drought (*p* <  0.01 except for 73-B; Fig. 6b). No increase in the percentage of deep roots was observed in 252-B as compared to Swarna. Therefore, dry mass analysis of the whole root system in lysimeters corroborated the field phenotypes adding evidence that decreased shallow and increased deep rooting were associated with the full segment of *qDTY*_*3.2*_.

### Water uptake in the field and lysimeters

**Fig. 7:**
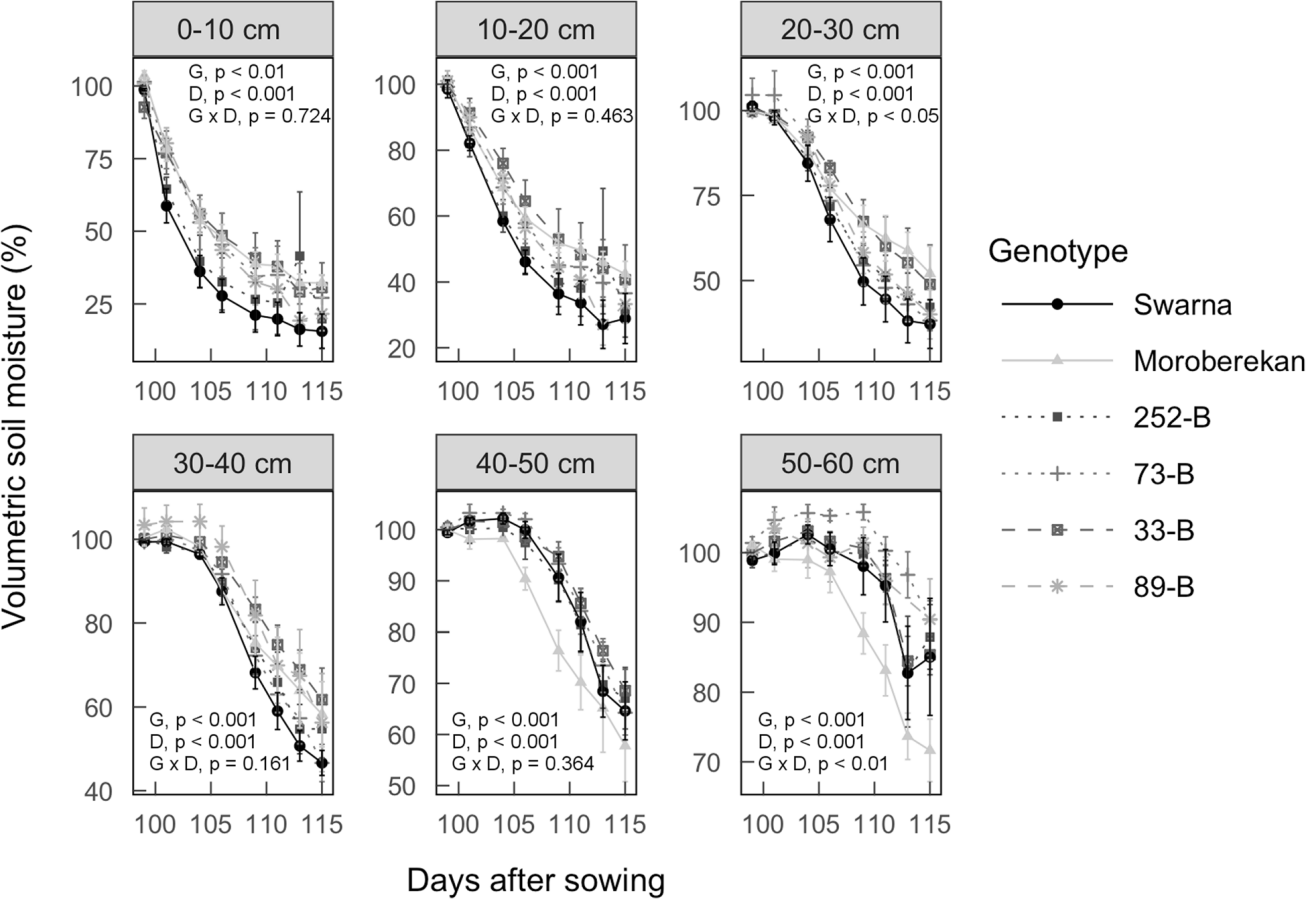
Reduction in volumetric soil moisture in the drought-stressed plots of Swarna, Moroberekan, and the four selected QTL lines in field Experiment 15DS. Soil moisture was expressed as percent of initial soil moisture after rewatering the field at 97 days after sowing. Mean values ± se (*n* = 4) are presented and *p*-values shown are for genotypic (G), dates (D) and genotype × date (G × D) differences across the different dates

To investigate the effect of *qDTY*_*3.2*_ associated RSA phenotypes on water uptake ability, changes in volumetric soil moisture at different depths were measured in plots planted with Swarna, Moroberekan, and the four selected QTL lines in the drought treatment of field Experiments 14DS and 15DS. In Experiment 15DS, soil planted with QTL lines and Moroberekan showed slower reduction in volumetric soil moisture as compared to soil planted with Swarna at depths of 0–40 cm (with *p* <  0.01 across dry-down period; Fig. 7). Below 40 cm, the volumetric soil moisture decreased more rapidly in soil planted with Moroberekan as compared to soil planted with the QTL lines and Swarna (with *p* <  0.001 across the dry-down period; Fig. 7). Despite a larger degree of variability, similar results were observed in Experiment 14DS (Additional file 10: Figure S9 and Additional file 11: Figure S10). These results indicate less water uptake by Moroberekan and the QTL lines as compared to Swarna in shallow soil layers and higher water uptake by Moroberekan in deeper soil layers.

Total water uptake (TWU) was also measured between 32 and 63 DAS in lysimeters (Additional file 1: Table S12). Under drought stress, when low differences in SDM were observed among genotypes, 73-B, 33-B and 89-B tended to show higher TWU as compared to 252-B and Swarna.

### Root system architecture measurements from the gel imaging platform

**Fig. 8:**
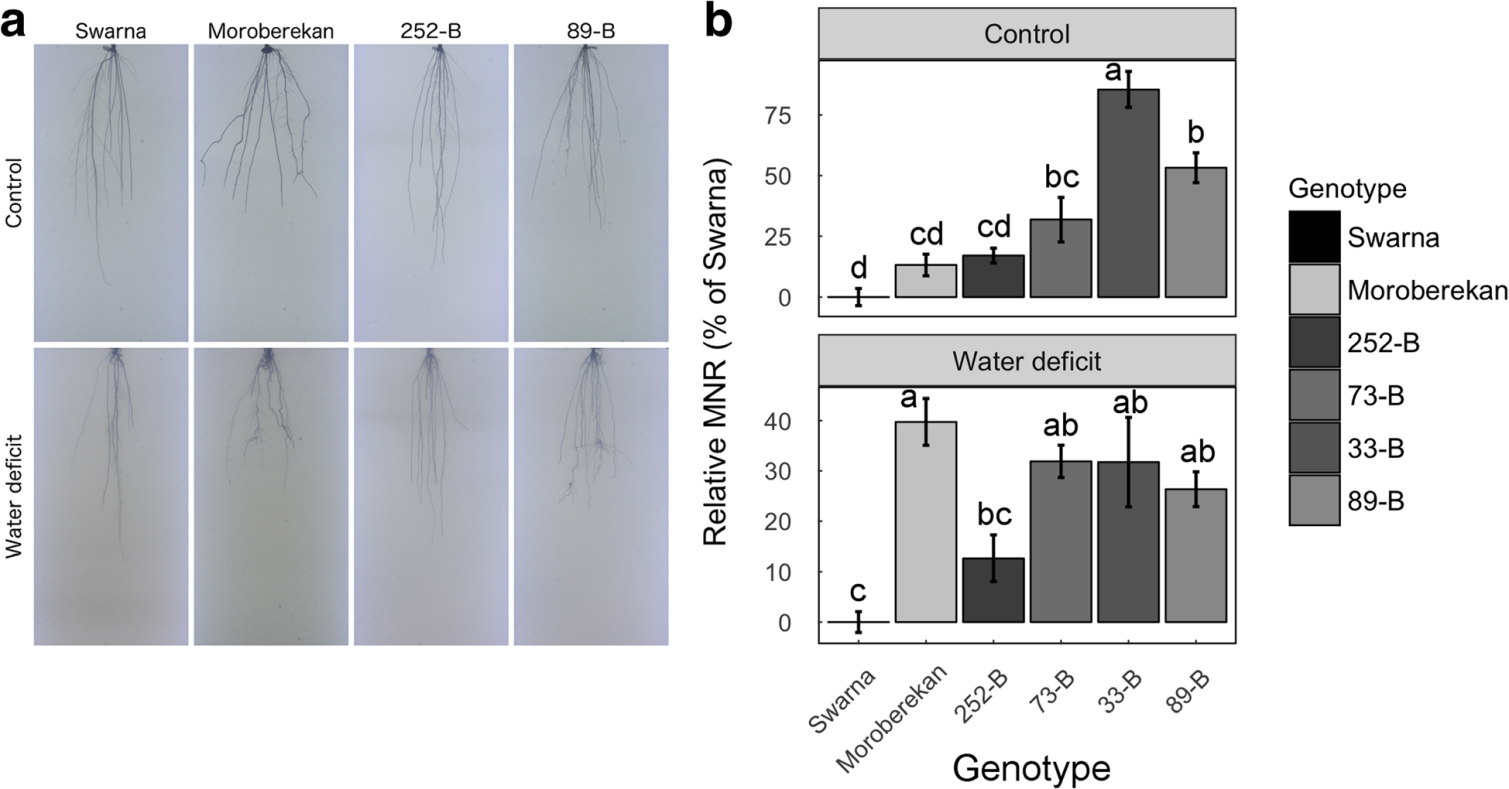
Root system architecture of Swarna, Moroberekan and the four selected QTL lines in a gel imaging system. Seedlings were grown in control (C: Yoshida) and water deficit (WD: Yoshida + 10% PEG) treatments and imaged at 15 days after germination for RSA analysis (**a**). Results of percent change in maximum number of roots (MNR) as compared to Swarna are presented (**b**). Bars represents mean values ± se (*n* = 26 for Swarna and Moroberekan, and *n* = 8–12 for the QTL lines) and letters indicate difference significance groups

To visualize and define RSA in more detail, Swarna, Moroberekan and the four selected QTL lines were grown in gel-filled cylinders under simulated well-watered (control) and water deficit (WD; 10% PEG) conditions, imaged at seedling stage (15 DAS; Fig. 8a and Additional file 12: Figure S11), and analyzed for RSA. Among the measured RSA traits, root width was observed to be higher in Moroberekan as compared to Swarna under both control and WD conditions (Additional file 1: Table S13). However, the QTL lines showed contrasting root widths, with 252-B and 73-B showing similar width to Swarna and 89-B showing similar width to Moroberekan under both conditions (*p* <  0.001). In addition, maximum number of roots (MNR, referring to the maximum number of roots) was higher in Moroberekan and the QTL lines as compared to Swarna under control and WD condition (Fig. 8b and Additional file 1: Table S13). In fact, the increase in percent MNR was significant in Moroberekan, 73-B, 33-B and 89-B as compared to Swarna under WD condition (*p* <  0.001). Overall, root phenotyping in gel at seedling stage showed that higher number of roots at seedling stage was associated with the presence of the full segment of *qDTY*_*3.2*_ under WD conditions.

## Discussion

Our study aimed to characterize the drought resistance physiological mechanisms associated with *qDTY*_*3.2*_ by phenotyping root and shoot drought resistance-related traits in fifteen BC_2_F_3:4_ lines with fixed *qDTY*_*3.2*_ introgressions in field conditions. We studied the physiological mechanisms associated with *qDTY*_*3.2*_ under severe drought stress that correspond to the conditions in which this QTL was identified (Dixit et al. 2014). These severe stress levels facilitated the distinction of drought-response traits from constitutive traits, while remaining within the scope of actual conditions occurring in rainfed lowland rice fields of South Asia where Swarna is a popular variety (Pandey et al. 2007). The QTL lines used in this study were derived from the original BC_2_ derived mapping population. Although *qDTY*_*3.2*_ was identified to be the only consistent major-effect QTL in this population and was the only known common genetic factor across all the lines under study (Dixit et al. 2014, 2015b), these lines were not *qDTY*_*3.2*_ NILs and there is a possibility of unknown introgressions from Moroberekan having an effect on the traits measured. To take this limitation into account, our approach was to select four QTL lines with contrasting yield under drought and look for phenotypes that were consistent across the four lines as compared to Swarna. Our hypothesis was that consistent phenotypes would be associated with *qDTY*_*3.2*_, while inconsistent phenotypes would be linked to the genetic background that may differentially influence the *qDTY*_*3.2*_-associated phenotypes. We further confirmed the *qDTY*_*3.2*_-associated phenotypes on a larger panel of lines.

### RSA traits associated with the presence of *qDTY*_*3.2*_

Correlation analyses suggested that root traits were important in drought-yield resistance of the *qDTY*_*3.2*_ lines although coefficients were <  0.5 in most cases (Table 2). Reduction in shallow root growth in the four selected QTL lines and Moroberekan was observed across the five field experiments and in the lysimeter study as compared to Swarna (Figs. 4a and 6a). The lower RLD in shallow soil layers of these QTL lines was further supported by the reduction in their crown root number as compared to Swarna (Additional file 1: Table S11). Concomitantly, increased deep root growth in the QTL lines and Moroberekan that appeared to be drought-induced was observed across the five field experiments and in the lysimeter study as compared to Swarna (except for 252-B with partial introgression of *qDTY*_*3.2*_; Fig. 4b and 6b), and was positively correlated with GY in at least three field experiments (Table 2). These results were further supported by a re-analysis of the root mass density along the soil profile obtained in a larger population of + *qDTY*_*3.2*_ and – *qDTY*_*3.2*_ lines (Fig. 5), and agree with the significant positive correlation between GY and RDM below a depth of 45 cm in the entire BC_2_F_3:4_ population (Dixit et al. 2015a). Overall, our results show that reduced shallow root growth and drought-induced increased deep root growth were consistently observed in QTL lines with full introgression of *qDTY*_*3.2*_, supporting the hypothesis that these RSA phenotypes were associated with the presence of *qDTY*_*3.2*_.

### Robustness of the *qDTY*_*3.2*_-associated RSA phenotypes

Root phenotyping in field conditions is challenging but remains the most reliable approach to understand the relationship between RSA and improved yield under drought. Soil coring has been widely used to investigate RSA of several crop species such as rice, wheat (*Triticum aestivum*), and maize (*Zea mays*) in field conditions (Swamy et al. 2013; Wasson et al. 2014; Wu and Guo 2014). However, these measurements are subjected to a high degree of variability within and between experiments due to strong environmental effects. Therefore, when screening for genotypic differences in RSA in field conditions, the most reliable phenotypes are those that are most consistent across several experiments and growth systems (Langridge and Reynolds 2015). In our study, the *qDTY*_*3.2*_-associated RSA phenotypes were observed in at least three of the five field experiments for each genotype and in lysimeters, suggesting that these phenotypes are robust. The physiological characterization of NILs for *qDTY*_*3.2*_ will allow confirmation and more precise description of the RSA traits that are associated with the presence of that QTL. In particular, it remains to be determined whether *qDTY*_*3.2*_ has a direct effect on reduced shallow root formation or if this phenotype is a consequence of faster maturation and relatively lower tiller number in the QTL lines as compared to Swarna (Additional file 1: Tables S4 and S10).

Root phenotyping in a gel imaging platform can provide additional information on the whole RSA, although it does not reflect the heterogeneous and mechanical properties of the soil. For instance, root growth in gel allows evaluation of 2- or 3-dimensional RSA traits that are lost when washing roots grown in soil. In this study, RSA analysis in the gel imaging platform revealed that the most drought-resistant QTL lines showed higher MNR (the maximum number of roots) as compared to Swarna (Fig. 8b). This phenotype suggests higher branching in the QTL lines which, when appearing at depth, may result in exploration of larger soil volumes. An apparent analogy could be viewed between higher MNR in the gel imaging platform and increased deep root growth in the field and lysimeters. Therefore, MNR might be a relevant trait to consider in gel imaging experiments aiming to identify root-related drought resistance traits useful in breeding programs. In wheat, seedling root traits correlated positively in the laboratory and field, but juvenile traits were not necessary correlated with adult root traits (Watt et al. 2013). New techniques that can visualize and quantify unaltered root systems in granular substrate or soil by X-Ray computed tomography or magnetic resonance imaging are being developed, and will allow better definition of RSA phenotypes at later developmental stages in field-related conditions (Metzner et al. 2015; Pfeifer et al. 2015; Rogers et al. 2016).

### Effect of *qDTY*_*3.2*_-associated RSA phenotypes on plant water uptake

The *qDTY*_*3.2*_-related RSA phenotypes observed in the QTL lines suggested that their water uptake abilities along the soil profile differed from Swarna under drought stress, with lower water uptake near the surface due to reduced root proliferation, and higher water uptake at depth due to increased soil exploration. Although slower reduction in volumetric soil moisture in plots planted with the QTL lines and Moroberekan was observed at shallow depths as compared to Swarna, no differences in volumetric soil moisture between the QTL lines and Swarna were observed below 30 cm (Fig. 7 and Additional file 10: Figure S9 and Additional file 11: Figure S10). These observations may reflect the limited measurement of just a narrow column of soil, rather than overall water capture of the whole root system that may well be higher in the QTL lines compared to Swarna. The latter hypothesis is supported by the higher TWU observed in 73-B, 33-B and 89-B as compared to Swarna under drought stress in lysimeters (Additional file 1: Table S12). Therefore, our results suggest that a combination of less root proliferation and water uptake near the soil surface, and higher root growth at depth (perhaps associated with higher deep water uptake), have contributed to better water budgeting under drought stress. Indeed, reduced root production in shallow soil layers under drought stress may have reduced metabolic cost of root production in shallow soil for inefficient water scavenging and subsequent production of roots where they are needed, i.e. in deeper soil (Lynch 2015). Similar strategies for drought-resistance were reported in maize and foxtail-millet (*Setaria italica*), where it is suggested that crown roots locally sense water deficit and suppress postemergence crown root growth, thus promoting rooting depth (Gao and Lynch 2016; Sebastian et al. 2016). Higher root proliferation at depth may be particularly beneficial for drought resistance in lowland soils to which Swarna is adapted, which tend to crack on the surface when drying, increasing soil water evaporation (Cairns et al. 2011). In the *qDTY*_*3.2*_ lines, further studies are needed to investigate if the higher percentage of deep root growth is due to higher lateral root density or increased lateral root length and clarify the role of specific root types in water uptake along the soil profile.

### Shoot-related traits associated with the presence of *qDTY*_*3.2*_

In addition to root traits, earlier time to flowering contributed to drought-yield resistance in the QTL lines (Table 2) and was consistently observed in Moroberekan and the QTL lines as compared to Swarna under drought stress but also well-watered conditions (Fig. 3). In fact, the QTL lines lacked a notable time to flowering delay by drought stress in contrast to Swarna. These results suggest that time to flowering in the QTL lines was most likely associated with the presence of *qDTY*_*3.2*_. However, since earlier time to flowering was expressed to different degrees in the QTL lines, this phenotype may also be influenced by the genetic background. Shoot morphological traits such as tiller number or SDM were variable among QTL lines and are therefore likely to be linked to the presence of additional introgressions independent of the presence of *qDTY*_*3.2*_. The lower canopy temperature observed in 33-B, 89-B, and Moroberekan contributed to their high drought-yield resistance as suggested by correlation analyses (Table 2). However, this trend was not strictly related to the presence of *qDTY*_*3.2*_ and may therefore also have been influenced by the genetic background.

### Effect of *qDTY*_*3.2*_-associated root and shoot phenotypes on grain yield

In our study, the yield increase in the *qDTY*_*3.2*_ lines was variable and low due to the severity of the drought stress (around 10% of the GY observed under well-watered conditions). Although shoot growth remained reasonable in the QTL lines under these severe drought stress conditions (33 to 73% of the SDM observed under well-watered conditions), HI was drastically reduced indicating poor resource allocation towards the grains at reproductive stage (Table 4). Notably, the identified traits associated with the presence of *qDTY*_*3.2*_, i.e. time to flowering and RSA phenotypes, were not always sufficient to increase yield under severe drought stress. In fact, we observed that the physiological traits associated with the presence of *qDTY*_*3.2*_ seem more stable than the yield advantage, which is in line with previously reported results on the characterization of *qDTY*_*12.1*_ (Henry et al. 2014). In lines 33-B and 89-B, it appears that rather than one trait alone, a combination of different traits including deep roots, earlier flowering time but also lower canopy temperature contributed to the significant improvement in yield under severe drought stress conditions as indicated by correlation analyses (Table 2). We hypothesize that, in the best-performing QTL lines, the *qDTY*_*3.2*_-related RSA phenotypes were able to sustain shoot water status that maintained low canopy temperature during a longer period of time than in Swarna. Interestingly, *qDTY*_*3.2*_ is part of a cluster co-locating with QTLs for time to flowering (*HD9*) and drought resistance related-traits such as index of canopy cover and canopy temperature (Dixit et al. 2015a). Therefore, different sizes of the *qDTY*_*3.2*_ introgression may also have resulted in different interactions of *qDTY*_*3.2*_ with the genetic background. The hypothesis that a large-effect drought-yield QTL in rice may function by multiple QTLs/genes within the QTL is supported by the characterization of *qDTY*_*12.1*_, where multiple intra-QTL genes regulated by the intra-QTL transcription factor *NO APICAL MERISTEM 12.1* (*OsNAM*_*12.1*_) induce multiple drought-response mechanisms including root branching (Dixit et al. 2015b).

## Conclusions

Since *qDTY*_*3.2*_ was previously genetically characterized to increase grain yield under drought in the Swarna background (Dixit et al. 2014; Dixit et al. 2015a), this study focused on physiological characterization of the QTL associated traits. The detailed characterization of BC_2_F_3:4_ lines generated from the cross between Swarna and Moroberekan in which the major drought yield QTL *qDTY*_*3.2*_ was fixed suggested three main phenotypes to be associated with this QTL: (1) decreased root proliferation near the soil surface, (2) drought-induced increased soil exploration at depth and (3) earlier time to flowering. The genotypic differences in root morphology were evidenced through measurements at seedling stage in a gel imaging system, at early vegetative stage in soil-filled lysimeters, and at maturity in the field. The *qDTY*_*3.2*_-associated RSA phenotypes were further confirmed in a larger +/− *qDTY*_*3.2*_ population (37/18 lines).

The QTL lines with highest yield under drought also showed lower canopy temperature that was not strictly associated with the presence of *qDTY*_*3.2*_, suggesting that the *qDTY*_*3.2*_-associated RSA traits may, in some cases, contribute to maintaining whole plant water status by interacting with shoot-related drought resistance traits. *qDTY*_*3.2*_ has been one of the most consistent QTLs across studies conducted on GY under drought at IRRI. NILs of this QTL in different genetic backgrounds with alleles from different sources are becoming available. This material can be used to further investigate the different genes/physiological mechanisms behind *qDTY*_*3.2*_, their interactions and their validity in different genotypes and drought scenarios. Ultimately, these studies will support the definition of appropriate drought-resistance QTLs to introgress into varieties that are well adapted to targeted drought-prone environments.

## Additional files

Additional file 1: **Table S1.** Genetic identity of the rice Moroberekan x Swarna- BC_2_F_3_ derived genotypes used in this study for physiological characterization. **Table S2.** Grain yield (kg ha^− 1^) of Swarna, Moroberekan, and the QTL lines in the well-watered treatment of field experiments. **Table S3.** Grain yield (kg ha^− 1^) of Swarna, Moroberekan, and the QTL lines in the drought stress treatment of field experiments. **Table S4.** Tiller number in field experiments. **Table S5.** Leaf area in Swarna, Moroberekan, and the selected QTL lines in the field. **Table S6.** Leaf width in Swarna, Moroberekan, and the selected QTL lines in the field. **Table S7.** Plant height in field experiments. **Table S8.** Shoot dry mass in field experiments. **Table S9.** Harvest index in Swarna, Moroberekan, and the selected QTL lines in the field. **Table S10.** Flowering time in field experiments. **Table S11.** Crown root number in Swarna, Moroberekan, and selected QTL lines in the field. **Table S12.** Shoot dry mass (SDM) and total water uptake (TWU) of Swarna, Moroberekan and the QTL lines in lysimeters. **Table S13.** Root system architecture (RSA) traits of Swarna, Moroberekan and QTL lines at seedling stage in the gel imaging platform.

Additional file 2: **Figure S1.** Representative images of Swarna, Moroberekan and the four selected lines (252-B, 73-B, 33-B and 89-B) in the field Experiment 13DS. In this experiment, plots consisted of three rows spaced at 0.25 m × 15 hills spaced at 0.20 m. The first row is indicated by the labelling stick. Images were taken at 88 days after sowing that corresponded to 13 days after the imposition of the drought stress. (PDF 26584 kb)

Additional file 3: **Figure S2.** Variation in canopy temperature of Swarna, Moroberekan, and the selected QTL lines in the drought stress treatment of Experiments 13DS, 13WS, 14DS and 15DS. In Experiments 13WS and 14DS, QTL lines were separated into two maturity groups (E: early and L: late). Mean values ± se (*n* = 4) are presented and *p*-values shown are for genotypic (G), dates (days after sowing: DAS) and genotype × date (G × DAS) differences for canopy temperature calculated across the different dates. (JPEG 1015 kb)

Additional file 4: **Figure S3.** Variation in maximum quantum efficiency (MQE, Fv/Fm) of photosystem II (PSII) in Swarna, Moroberekan, and the selected QTL lines in the drought stress treatment of field experiments. Fv/Fm was typically measured between 9 AM to 11 PM at sunny and not windy times using a Handy Pea chlorophyll fluorometer (Hansatech Instruments Ltd., England) at different dates during soil dry-down. Two fully expanded leaves from 2 different plants were used per plot (flag leaves were excluded). The leaf area subjected to the measurement was dark-adapted for at least 30 min using leaf clips of 4 mm diameter firmly attached to bamboo sticks. In Experiment 13WS, QTL lines were separated into two maturity groups (E: early and L: late). Mean values ± se (*n* = 4) are presented. (JPEG 658 kb)

Additional file 5: **Figure S4.** Carbon isotope discrimination (Δ^13^C) in Swarna, Moroberekan and the selected QTL lines in Experiment 15DS. Δ^13^C was measured on the youngest fully-formed leaves collected at 85 and 95 DAS (days after sowing) in both well-watered and drought stress treatments of Experiment 15DS using the following formula: (− 8 – leaf ^13^C concentration) / [1 + (leaf ^13^C concentration/1000)] (Farquhar et al. 1989). Bars show mean ± se (*n* = 4). (JPEG 401 kb)

Additional file 6: **Figure S5.** Stomatal density of Swarna, Moroberekan, and QTL lines in the field. Stomatal density was measured during the reproductive stage following the procedure of Kusumi et al. (2012). Epidermal imprints of the adaxial surface of three fully expanded leaves per plot were collected and the stomata present in an area of about 0.3 mm^2^ were counted under a microscope at 100× magnification. In Experiment 14DS, QTL lines were separated into two maturity groups (E: early and L: late). Bars show mean ± se (*n* = 4) and letters indicate significant difference groups within treatments. (JPEG 376 kb)

Additional file 7: **Figure S6.** Root length density (RLD) of Swarna, Moroberekan and the four selected QTL lines under drought stress conditions in the field. Bars represent mean root length density (*n* = 3–4) from 0 to 30 cm (A) and 30–60 cm (B) of each experiment. Results of RLD for Swarna and Moroberekan grown in the late maturity group of Experiments 13WS and 14DS are presented. *P*-values shown are for genotypic (G), experimental (E) and genotype × experiment (G × E) differences for RLD calculated across the different experiments. Letters indicate different significance groups. (JPEG 574 kb)

Additional file 8: **Figure S7.** Root length density (RLD) of Swarna, Moroberekan and the four selected QTL lines under well-watered conditions in the field. Bars represent mean root length density (*n* = 4) from 0 to 30 cm (A) and 30–60 cm (B) of each experiment. RLD was not measured in the well-watered treatment of Experiment 13DS. Results of RLD for Swarna and Moroberekan grown in the late maturity group of Experiments 13WS and 14DS are presented. *P*-values shown are for genotypic (G), experimental (E) and genotype × experiment (G × E) differences for RLD calculated across the different experiments. Letters indicate different significance groups. (JPEG 541 kb)

Additional file 9: **Figure S8.** Percentage of shallow and deep root length in Swarna, Moroberekan and the four selected QTL lines under well-watered conditions in the field. Total root length measured in 15-cm soil segments from 0 to 60 cm were analyzed to calculate percent (%) shallow (from 0 to 30 cm; A) and deep (from 30 to 60 cm; B) roots. Total root length was not measured in the well-watered treatment of Experiment 13DS. Results from the late maturity group of Experiments 13WS and 14DS are presented for Swarna and Moroberekan. Bars represent mean values ± se (*n* = 4). P-values shown are for genotypic (G), experimental (E) and genotype × experiment (G × E) differences calculated across the different experiments. (JPEG 511 kb)

Additional file 10: **Figure S9.** Variations of volumetric soil moisture in the drought-stressed plots of Swarna, Moroberekan, 33-B, 89-B in Experiment 14DS (E). Soil moisture was expressed as percent of initial soil moisture after initiation of the drought stress at 60 days after sowing (DAS). Mean values ± se (*n* = 4) are presented and *p*-values shown are for genotypic (G), dates (DAS) and genotype × date (G × DAS) differences for volumetric soil moisture calculated across the different dates. (JPEG 1034 kb)

Additional file 11: **Figure S10.** Variations of volumetric soil moisture in the drought-stressed plots of Swarna, Moroberekan, 252-B and 73-B in Experiment 14DS (L). Soil moisture was expressed as percent of initial soil moisture after initiation of the drought stress at 70 days after sowing (DAS). Mean values ± se (*n* = 4) are presented and p-values shown are for genotypic (G), dates (DAS) and genotype × date (G × DAS) differences for volumetric soil moisture calculated across the different dates. (JPEG 941 kb)

Additional file 12: **Figure S11.** Representative images of the root system of Swarna, Moroberekan, and the QTL lines grown in the gel imaging platform. Seedlings grown under control (Yoshida) or water deficit (Yoshida + PEG 10%) conditions were imaged at 15 days after germination or 12 days after transplanting. (JPEG 600 kb)

## Abbreviations

C: Control
CT: Canopy temperature
DAS: Days after sowing
DS: Dry season
DTF: Time to flowering
E: Early maturity group
GY: Grain yield
HI: Harvest index
L: Late maturity group
MNR: Maximum number of roots
NILs: Near isogenic lines
PEG: Polyethylene glycol 8000
QTL: Quantitative trait locus
RDM: Root dry mass
RLD: Root length density
RSA: Root system architecture
SA: Surface area
SDM: Shoot dry mass
TL: Total root length
TWU: Total water uptake
WD: Water deficit
WS: Wet season
