# Supplementary material for: Physiological mechanisms contributing to the QTL qDTY3.2 effects on improved performance of rice Moroberekan x Swarna BC2F3:4 lines under drought
Source: Rice (N Y). 2018 Jul 31;11:43. doi: 10.1186/s12284-018-0234-1 (PMC6068063; doi:10.1186/s12284-018-0234-1)
Supplement: Supplementary file 1 — Table S1. Genetic identity of the rice Moroberekan x Swarna- BC2F3 derived genotypes used in this study for physiological characterization. Table S2. Grain yield (kg ha− 1) of Swarna, Moroberekan, and the QTL lines in the well-watered treatment of field experiments. Table S3. Grain yield (kg ha− 1) of Swarna, Moroberekan, and the QTL lines in the drought stress treatment of field experiments. Table S4. Tiller number in field experiments. Table S5. Leaf area in Swarna, Moroberekan, and the selected QTL lines in the field. Table S6. Leaf width in Swarna, Moroberekan, and the selected QTL lines in the field. Table S7. Plant height in field experiments. Table S8. Shoot dry mass in field experiments. Table S9. Harvest index in Swarna, Moroberekan, and the selected QTL lines in the field. Table S10. Flowering time in field experiments. Table S11. Crown root number in Swarna, Moroberekan, and selected QTL lines in the field. Table S12. Shoot dry mass (SDM) and total water uptake (TWU) of Swarna, Moroberekan and the QTL lines in lysimeters. Table S13. Root system architecture (RSA) traits of Swarna, Moroberekan and QTL lines at seedling stage in the gel imaging platform. [file 12284_2018_234_MOESM1_ESM.docx]

**Physiological mechanisms contributing to the QTL *qDTY_3.2_* effects on improved performance of rice Moroberekan x Swarna BC_2_F_3:4_ lines under drought**

Alexandre Grondin^1,^**^¶^**, Shalabh Dixit^1^, Rolando Torres^1^, Challa Venkateshwarlu^1,2^, Eric Rogers^2^, Thomas Mitchell-Olds^2^, Philip N. Benfey^2^, Arvind Kumar^1,2^ and Amelia Henry^1*^

*^1^International Rice Research Institute, Los Baños, Laguna, Philippines; ^2^International Rice Research Institute South Asia Hub, ICRISAT, Patancheru, Telangana, India; ^3^ Duke University, Department of Biology and Howard Hughes Medical Institute, Durham, NC, USA*

**^¶^***Present address:* *UMR DIADE, Institut de Recherche pour le Développement/Université de Montpellier, Montpellier, France*

**For correspondence. E-mail a.henry@irri.org*

| **Table S1** Genetic identity of the rice Moroberekan x Swarna- BC_2_F_3_ derived genotypes used in  this study for physiological characterization. | | | | | |
| --- | --- | --- | --- | --- | --- |
| Genotype ID | QTL line | *qDTY_3.2_* | *Maturity*  *group* |  | Experiment |
|  |  |  |  |  |  |
| ***Parents*** |  |  |  |  |  |
| Swarna |  | − | E, L |  | Grown as control lines in all  experiments except in field Experiment  13WS (E) for Moroberekan |
| Moroberekan |  | + | E, L |  |  |
|  |  |  |  |  |  |
| ***Selected BC_2_F_3:4_ lines*** | | | | | |
| IR 91648-B-252-B | 252-B | PARTIAL | L |  | Grown for physiological  characterization in field  Experiments 13DS, 13WS, 14DS,  14WS and 15DS, and for RSA  characterization in lysimeter and  gel imaging platform |
| IR 91648-B-33-B | 33-B | + | E |  |  |
| IR 91648-B-73-B | 73-B | + | L |  |  |
| IR 91648-B-89-B | 89-B | + | E |  |  |
|  |  |  |  |  |  |
| ***BC_2_F_3:4_ lines*** | | | | | |
| IR 91648-B-177-B | 177-B | PARTIAL | L |  | Grown for physiological  characterization in field  Experiments 13DS, 13WS  and 14DS, and in field Experiment  14WS for lines 245-B, 82-B and 87-B |
| IR 91648-B-182-B | 182-B | + | L |  |  |
| IR 91648-B-192-B | 192-B | PARTIAL | L |  |  |
| IR 91648-B-23-B | 23-B | + | E |  |  |
| IR 91648-B-245-B | 245-B | + | L |  |  |
| IR 91648-B-298-B | 298-B | + | E |  |  |
| IR 91648-B-319-B | 319-B | + | L |  |  |
| IR 91648-B-65-B | 65-B | + | L |  |  |
| IR 91648-B-82-B | 82-B | + | E |  |  |
| IR 91648-B-87-B | 87-B | + | E |  |  |
| IR 91648-B-95-B | 95-B | + | E |  |  |
|  |  |  |  |  |  |
| ***BC_2_F_3:6_ lines*** | | | | | |
| IR 91648-B-59-B-3-1 | 59-B-3-1 | + | n.a. |  | Grown for physiological  characterization in field  Experiment 14WS, and in  Experiment 15DS for lines  238-B-2-1 and 89-B-10-1 |
| IR 91648-B-182-B-1-B | 182-B-1-B | + | n.a. |  |  |
| IR 91648-B-238-B-2-1 | 238-B-2-1 | + | n.a. |  |  |
| IR 91648-B-319-B-2-1 | 319-B-2-1 | + | n.a. |  |  |
| IR 91648-B-89-B-10-1 | 89-B-10-1 | + | n.a. |  |  |
|  |  |  |  |  |  |
| The mapping population and identification of the drought tolerant QTL *qDTY_3.2_* is described in  Dixit et al. (2014). −: absence of *qDTY_3.2_*. +: Presence of the complete segment of *qDTY_3.2_*.  PARTIAL: Presence of partial segment of *qDTY_3.2_* with positive peak marker. In Experiments  13WS and 14DS QTL lines were separated into two maturity groups (E: early and L: late).  n.a.: not applicable because genotype not included in the Experiments 13WS and 14DS. | | | | | |

| **Table S2** Grain yield (kg ha^-1^) of Swarna, Moroberekan, and the QTL lines in the well-watered treatment of field experiments. | | | | | | | | | |
| --- | --- | --- | --- | --- | --- | --- | --- | --- | --- |
| Genotype |  | Experiment | | | | | | |  |
|  |  | 13DS | 13WS (E) | 13WS (L) | 14DS (E) | 14DS (L) | 14WS | 15DS |  |
|  |  |  |  |  |  |  |  |  |  |
| Swarna |  | 5550±331 | 1718±240 | 2223±409 | 3993±595 | 5207±495 | 4186±162 | 5292±611 |  |
| Moroberekan |  | 3212±179 | n.a. | 2329±111 | 2250±186 | 2567±313 | 2704±223 | 2795±190 |  |
| 252-B |  | 4942±257 | n.a. | 2367±324 | n.a. | 4118±343 | 3528±345 | 4704±355 |  |
| 73-B |  | 5866±643 | n.a. | 2935±307 | n.a. | 4476±275 | 4554±316 | 5602±478 |  |
| 33-B |  | 4818±190 | 2800±328 | n.a. | 2575±147 | n.a. | 4138±554 | 4106±487 |  |
| 89-B |  | 5590±271 | 3288±436 | n.a. | 4203±172 | n.a. | 4491±303 | 5428±412 |  |
|  |  |  |  |  |  |  |  |  |  |
| 177-B |  | 4330±229 | n.a. | 2336±119 | n.a. | 2968±257 | n.a. | n.a. |  |
| 182-B |  | 6346±227 | n.a. | 3716±340 | n.a. | 3825±345 | n.a. | n.a. |  |
| 192-B |  | 3641±228 | n.a. | 2331±312 | n.a. | 2901±125 | n.a. | n.a. |  |
| 23-B |  | 5957±351 | 2682±164 | n.a. | 3844±209 | n.a. | n.a. | n.a. |  |
| 245-B |  | 4789±378 | n.a. | 2881±243 | n.a. | 3671±356 | 3886±184 | n.a. |  |
| 298-B |  | 4593±259 | 2538±342 | n.a. | 2146±149 | n.a. | n.a. | n.a. |  |
| 319-B |  | 5711±425 | n.a. | 3662±68 | n.a. | 4397±167 | n.a. | n.a. |  |
| 65-B |  | 5093±240 | n.a. | 3468±289 | n.a. | 3406±290 | n.a. | n.a. |  |
| 82-B |  | 4555±335 | 2653±298 | n.a. | 3307±59 | n.a. | 4123±868 | n.a. |  |
| 87-B |  | 3442±134 | 2007±282 | n.a. | 2999±93 | n.a. | 4062±244 | n.a. |  |
| 95-B |  | 5726±324 | 2849±182 | n.a. | 3762±162 | n.a. | n.a. | n.a. |  |
|  |  |  |  |  |  |  |  |  |  |
| 59-B-3-1 |  | n.a. | n.a. | n.a. | n.a. | n.a. | 3317±275 | n.a. |  |
| 182-B-1-B |  | n.a. | n.a. | n.a. | n.a. | n.a. | 4017±308 | n.a. |  |
| 238-B-2-1 |  | n.a. | n.a. | n.a. | n.a. | n.a. | 4299±494 | 5433±214 |  |
| 319-B-2-1 |  | n.a. | n.a. | n.a. | n.a. | n.a. | 3722±492 | n.a. |  |
| 89-B-10-1 |  | n.a. | n.a. | n.a. | n.a. | n.a. | 3721±289 | 5969±279 |  |
|  |  |  |  |  |  |  |  |  |  |
| *Genotype (p-value)* |  | *< 0.001* | *< 0.05* | *< 0.001* | *< 0.001* | *< 0.001* | *0.578* | *< 0.001* |  |
| In Experiments 13WS and 14DS QTL lines were separated into two maturity groups (E: early and L: late). Mean values ± se (n=4) are presented.  n.a.: not applicable because genotype not included in the experiment. | | | | | | | | | |

| **Table S3** Grain yield (kg ha^-1^) of Swarna, Moroberekan, and the QTL lines in the drought stress treatment of field experiments. | | | | | | | | |
| --- | --- | --- | --- | --- | --- | --- | --- | --- |
| Genotype |  | Experiment | | | | | | |
|  |  | 13DS | 13WS (E) | 13WS (L) | 14DS (E) | 14DS (L) | 14WS | 15DS |
|  |  |  |  |  |  |  |  |  |
| Swarna |  | 50±9 | 143±53 | 437±153 | 3±3 | 9±9 | 1879±337 | 188±20 |
| Moroberekan |  | 94±14 | n.a. | 808±30 | 519±86 | 744±167 | 1416±250 | 399±55 |
| 252-B |  | 85±17 | n.a. | 271±44 | n.a. | 85±45 | 1740±161 | 310±60 |
| 73-B |  | 116±13 | n.a. | 105±34 | n.a. | 86±13 | 2684±218 | 349±41 |
| 33-B |  | 599±60 | 701±221 | n.a. | 78±23 | n.a. | 2642±166 | 523±138 |
| 89-B |  | 597±37 | 228±82 | n.a. | 77±16 | n.a. | 2169±302 | 437±153 |
|  |  |  |  |  |  |  |  |  |
| 177-B |  | 66±8 | n.a. | 96±30 | n.a. | 95±22 | n.a. | n.a. |
| 182-B |  | 128±13 | n.a. | 440±109 | n.a. | 283±79 | n.a. | n.a. |
| 192-B |  | 18±6 | n.a. | 121±95 | n.a. | 62±16 | n.a. | n.a. |
| 23-B |  | 278±25 | 380±154 | n.a. | 26±15 | n.a. | n.a. | n.a. |
| 245-B |  | 177±24 | n.a. | 434±90 | n.a. | 102±41 | 2153±211 | n.a. |
| 298-B |  | 194±26 | 174±63 | n.a. | 152±89 | n.a. | n.a. | n.a. |
| 319-B |  | 86±14 | n.a. | 388±66 | n.a. | 143±59 | n.a. | n.a. |
| 65-B |  | 164±17 | n.a. | 887±310 | n.a. | 305±60 | n.a. | n.a. |
| 82-B |  | 142±14 | 108±17 | n.a. | 4±4 | n.a. | 1477±87 | n.a. |
| 87-B |  | 647±63 | 358±178 | n.a. | 50±21 | n.a. | 2812±387 | n.a. |
| 95-B |  | 129±15 | 412±120 | n.a. | 27±27 | n.a. | n.a. | n.a. |
|  |  |  |  |  |  |  |  |  |
| 59-B-3-1 |  | n.a. | n.a. | n.a. | n.a. | n.a. | 2285±254 | n.a. |
| 182-B-1-B |  | n.a. | n.a. | n.a. | n.a. | n.a. | 2265±105 | n.a. |
| 238-B-2-1 |  | n.a. | n.a. | n.a. | n.a. | n.a. | 2434±286 | 487±212 |
| 319-B-2-1 |  | n.a. | n.a. | n.a. | n.a. | n.a. | 2225±95 | n.a. |
| 89-B-10-1 |  | n.a. | n.a. | n.a. | n.a. | n.a. | 1667±78 | 521±161 |
|  |  |  |  |  |  |  |  |  |
| *Genotype (p-value)* |  | *< 0.001* | *0.062* | *< 0.001* | *< 0.001* | *< 0.001* | *< 0.01* | *0.673* |
| In Experiments 13WS and 14DS QTL lines were separated into two maturity groups (E: early and L: late). Mean values ± se (n=4) are presented.  n.a.: not applicable because genotype not included in the experiment. | | | | | | | | |

| **Table S4** Tiller number (plant^-1^) in field experiments. | | | | | | | | |
| --- | --- | --- | --- | --- | --- | --- | --- | --- |
| Treatment | Genotype | Experiment | | | | | | |
|  |  | 13DS | 13WS (E) | 13WS (L) | 14DS (E) | 14DS (L) | 14WS | 15DS |
|  |  |  |  |  |  |  |  |  |
| Well-watered | Swarna | 31±4 a | 14±1 a | 14±1 a | 21±1 a | 18 a | 22±1 a | 24±5 |
|  | Moroberekan | 18±1 b | n.a. | 6 b | 5 c | 8±1 b | 8±1 c | 18 |
|  | 252-B | 25±2 ab | n.a. | 12±1 a | n.a. | 16±1 a | 19±2 ab | 21±4 |
|  | 73-B | 18±2 b | n.a. | 10 a | n.a. | 14±2 a | 15±1 b | 24±3 |
|  | 33-B | 15±3 b | 14±1 a | n.a. | 12±1 b | n.a. | 15±1 b | 17±1 |
|  | 89-B | 22±3 ab | 11 b | n.a. | 13 b | n.a. | 14±1 b | 12±1 |
|  |  |  |  |  |  |  |  |  |
| *Genotype (p-value)* | | *< 0.01* | *< 0.05* | *< 0.001* | *< 0.001* | *< 0.01* | *< 0.001* | *0.07* |
|  | |  |  |  |  |  |  |  |
| Drought stress | Swarna | 23±4 | 15±1 | 14±1 a | 18±2 a | 17±2 a | 22±3 a | 17±1 |
|  | Moroberekan | 11±2 | n.a. | 6 b | 6±1 c | 5±1 b | 9±1 b | 11±1 |
|  | 252-B | 15±3 | n.a. | 14±1 a | n.a. | 15±1 a | 21±2 a | 17±2 |
|  | 73-B | 18±3 | n.a. | 12±1 a | n.a. | 13±2 a | 17±1 a | 17±3 |
|  | 33-B | 23±3 | 13±2 | n.a. | 12±1 b | n.a. | 16±2 ab | 12±3 |
|  | 89-B | 26±7 | 15±1 | n.a. | 13±1 b | n.a. | 16±1 a | 14±1 |
|  |  |  |  |  |  |  |  |  |
| *Genotype (p-value)* | | *0.11* | *0.63* | *< 0.001* | *< 0.001* | *< 0.01* | *< 0.001* | *0.22* |
| In Experiments 13WS and 14DS, QTL lines were separated into two maturity groups (E: early and L: late). Mean values ± se (n=4) are presented. Letters indicate significant difference groups within a particular treatment of each experiment. n.a.: not applicable because the genotype was not included in the experiment. | | | | | | | | |

| **Table S5** Leaf area (cm^2^) in Swarna, Moroberekan, and the selected QTL lines in the field. | | | | | | |
| --- | --- | --- | --- | --- | --- | --- |
| Treatment | Genotype | Experiment | | | | |
|  |  | 13WS (E) | 13WS (L) | 14DS (E) | 14DS (L) | 15DS |
|  |  |  |  |  |  |  |
| Well-watered | Swarna | 954±68 | 1902±189 | 524±108 | 1165±208 | 1833 ± 153 a |
|  | Moroberekan | n.a. | 1388±71 | 511±125 | 701±98 | 1296±28 ab |
|  | 252-B | n.a. | 1291±103 |  | 975±231 | 1261±100 b |
|  | 73-B | n.a. | 1761±216 |  | 1043±243 | 1563±161 ab |
|  | 33-B | 1640±267 | n.a. | 607±141 | n.a. | 1581±139 ab |
|  | 89-B | 1377±160 | n.a. | 663±84 | n.a. | 1355±131 ab |
|  |  |  |  |  |  |  |
| *Genotype (p-value)* | | *0.073* | *0.051* | *0.770* | *0.454* | *< 0.05* |
|  | |  |  |  |  |  |
| Drought stress | Swarna | 1323±275 | 1172±382 | 391±27 | 112±9 | 1359±193 |
|  | Moroberekan | n.a. | 774±226 | 367±58 | 181±71 | 1556±352 |
|  | 252-B | n.a. | 1074±180 |  | 172±36 | 1619±227 |
|  | 73-B | n.a. | 977±321 |  | 192±46 | 1287±157 |
|  | 33-B | 1792±143 | n.a. | 427±37 | n.a. | 1295±146 |
|  | 89-B | 1211±172 | n.a. | 424±47 | n.a. | 2106±513 |
|  |  |  |  |  |  |  |
| *Genotype (p-value)* | | *0.159* | *0.792* | *0.742* | *0.632* | *0.388* |
| In Experiments 13WS and 14DS QTL lines were separated into two maturity groups (E: early and L: late). Total leaf area (cm^2^) was measured  at 65, 96, 73, 93 and 72 days after sowing in Experiments 13WS (E), 13WS (L), 14DS (E), 14DS (L) and 15DS, respectively. Mean values ± se (n=4)  are presented. Letters indicate significant difference groups within a particular treatment of each experiment. n.a.: not applicable because the  genotype was not included in the experiment. | | | | | | |

| **Table S6** Leaf width (cm) in Swarna, Moroberekan, and the selected QTL lines in the field. | | | | | | |
| --- | --- | --- | --- | --- | --- | --- |
| Treatment | Genotype | Experiment | | | | |
|  |  | 13WS (E) | 13WS (L) | 14DS (E) | 14DS (L) | 15DS |
|  |  |  |  |  |  |  |
| Well-watered | Swarna | 1.3 | 2.3 ab | 1.0±0.1 | 1.6±0.1 | 1.8±0.1 b |
|  | Moroberekan | n.a. | 2.7±0.1 a | 1.2±0.2 | 1.8±0.2 | 2.3±0.1 a |
|  | 252-B | n.a. | 2.1±0.1 b | n.a. | 1.5±0.1 | 1.8 b |
|  | 73-B | n.a. | 2.6±0.1 a | n.a. | 2.1±0.2 | 2.0±0.1 ab |
|  | 33-B | 1.5±0.2 | n.a. | 1.3±0.1 | n.a. | 2.1±0.1 ab |
|  | 89-B | 1.3 | n.a. | 1.3±0.1 | n.a. | 1.9±0.1 ab |
|  |  |  |  |  |  |  |
| *Genotype (p-value)* | | *0.177* | *< 0.01* | *0.327* | *0.100* | *< 0.05* |
|  | |  |  |  |  |  |
| Drought stress | Swarna | 1.3±0.1 b | 1.7±0.1 | 0.9 | 0.6±0.1 | 1.6±0.1 |
|  | Moroberekan | n.a. | 2.1±0.3 | 1.1±0.1 | 0.8±0.2 | 2.1±0.2 |
|  | 252-B | n.a. | 1.9±0.1 | n.a. | 0.7±0.1 | 1.8±0.1 |
|  | 73-B | n.a. | 1.7±0.2 | n.a. | 0.7±0.1 | 2±0.1 |
|  | 33-B | 1.6±0.1 a | n.a. | 1.2±0.1 | n.a. | 1.9±0.2 |
|  | 89-B | 1.6±0.1 ab | n.a. | 1.1±0.1 | n.a. | 2.1±0.2 |
|  |  |  |  |  |  |  |
| *Genotype (p-value)* | | *< 0.05* | *0.473* | *0.402* | *0.796* | *0.257* |
| In Experiments 13WS and 14DS QTL lines were separated into two maturity groups (E: early and L: late). Average leaf width was measured  at 65, 96, 73, 93 and 72 days after sowing in Experiments 13WS (E), 13WS (L), 14DS (E), 14DS (L) and 15DS, respectively. Mean values ± se (n=4)  are presented. Letters indicate significant difference groups within a particular treatment of each experiment. n.a.: not applicable because the genotype  was not included in the experiment. | | | | | | |

| **Table S7** Plant height (cm) in field experiments. | | | | | | | | |
| --- | --- | --- | --- | --- | --- | --- | --- | --- |
| Treatment | Genotype | Experiment | | | | | | |
|  |  | 13DS | 13WS (E) | 13WS (L) | 14DS (E) | 14DS (L) | 14WS | 15DS |
|  |  |  |  |  |  |  |  |  |
| Well-watered | Swarna | 87±1 bc | 105±2 b | 98±5 c | 73±2 c | 76±2 b | 102±2 bc | 92±4 c |
|  | Moroberekan | 92±5 b | n.a. | 140±3 a | 108±2 a | 117±5 a | 143±6 a | 132±4 a |
|  | 252-B | 80±2 c | n.a. | 92±2 c | n.a. | 71±3 b | 97±3 c | 77±2 d |
|  | 73-B | 93±2 b | n.a. | 118±5 b | n.a. | 78±2 b | 114±1 b | 92±1 c |
|  | 33-B | 119±3 a | 141±6 a | n.a. | 97±4 ab | n.a. | 146±3 a | 117±2 b |
|  | 89-B | 94±2 b | 118±3 b | n.a. | 87±2 b | n.a. | 136±3 a | 96 c |
|  |  |  |  |  |  |  |  |  |
| *Genotype (p-value)* | | *< 0.001* | *< 0.001* | *< 0.001* | *< 0.001* | *< 0.001* | *< 0.001* | *< 0.001* |
|  | |  |  |  |  |  |  |  |
| Drought stress | Swarna | 45±4 b | 73±7 b | 79±1 b | 45±5 c | 48±2 c | 87±2 b | 65±1 c |
|  | Moroberekan | 75±12 a | n.a. | 104±3 a | 90±1 a | 92±4 a | 128±5 a | 101±1 a |
|  | 252-B | 44±2 b | n.a. | 72±1 c | n.a. | 46±1 c | 83±2 b | 64±1 c |
|  | 73-B | 57±3 ab | n.a. | 85±1 b | n.a. | 62±1 b | 94±4 b | 71±1 c |
|  | 33-B | 75±2 a | 105±5 a | n.a. | 75±1 b | n.a. | 129±9 a | 99±2 a |
|  | 89-B | 70±1 a | 93±11 ab | n.a. | 68±2 b | n.a. | 117±2 a | 87±4 b |
|  |  |  |  |  |  |  |  |  |
| *Genotype (p-value)* | | *< 0.001* | *< 0.05* | *< 0.001* | *< 0.001* | *< 0.001* | *< 0.001* | *< 0.001* |
| In Experiments 13WS and 14DS, QTL lines were separated into two maturity groups (E: early and L: late). Mean values ± se (n=4) are presented.  Letters indicate significant difference groups within a particular treatment of each experiment. n.a.: not applicable because the genotype was not  included in the experiment. | | | | | | | | |

| **Table S8** Shoot dry mass (g m^-2^) in field experiments. | | | | | | | |
| --- | --- | --- | --- | --- | --- | --- | --- |
| Treatment | Genotype | Experiment | | | | | |
|  |  | 13DS | 13WS (E) | 13WS (L) | 14DS (E) | 14DS (L) | 14WS |
|  |  |  |  |  |  |  |  |
| Well-watered | Swarna | 1339±54 a | 791±135 | 828±44 a | 591±37 a | 603±15 | 821±50 |
|  | Moroberekan | 1122±58 ab | n.a. | 637±42 b | 436±10 b | 612±43 | 724±46 |
|  | 252-B | 1160±27 ab | n.a. | 622±34 b | n.a. | 522±38 | 843±32 |
|  | 73-B | 1270±31 ab | n.a. | 731±9 ab | n.a. | 597±38 | 800±61 |
|  | 33-B | 1056±106 b | 709±34 | n.a. | 404±22 b | n.a. | 813±27 |
|  | 89-B | 1244±23 ab | 717±22 | n.a. | 405±28 b | n.a. | 766±14 |
|  |  |  |  |  |  |  |  |
| *Genotype (p-value)* | | *< 0.05* | *0.74* | *< 0.01* | *< 0.001* | *0.29* | *0.19* |
|  | |  |  |  |  |  |  |
| Drought stress | Swarna | 436±23 | 569±77 | 525±21 | 247±12 | 328±34 | 893±37 |
|  | Moroberekan | 482±51 | n.a. | 432±87 | 243±13 | 357±29 | 765±56 |
|  | 252-B | 406±17 | n.a. | 455±43 | n.a. | 304±37 | 829±22 |
|  | 73-B | 413±35 | n.a. | 487±22 | n.a. | 369±26 | 904±44 |
|  | 33-B | 467±35 | 490±43 | n.a. | 297±22 | n.a. | 773±35 |
|  | 89-B | 454±26 | 542±14 | n.a. | 270±18 | n.a. | 876±78 |
|  |  |  |  |  |  |  |  |
| *Genotype (p-value)* | | *0.54* | *0.56* | *0.62* | *0.13* | *0.49* | *0.28* |
| In Experiments 13WS and 14DS, QTL lines were separated into two maturity groups (E: early and L: late).  Shoot dry mass (SDM) was measured at maturity. Mean values ± se (n=4) are presented. Letters indicate significant  difference groups within a particular treatment of each experiment. n.a.: not applicable because the genotype  was not included in the experiment. | | | | | | | |

| **Table S9** Harvest index in Swarna, Moroberekan, and the selected QTL lines in the field. | | | | | | | |
| --- | --- | --- | --- | --- | --- | --- | --- |
| Treatment | Genotype | Experiment | | | | | |
|  |  | 13DS | 13WS (E) | 13WS (L) | 14DS (E) | 14DS (L) | 14WS |
|  |  |  |  |  |  |  |  |
| Well-watered | Swarna | 0.29±0.01 a | 0.18±0.01 b | 0.21±0.02 | 0.40±0.03 b | 0.46±0.02 a | 0.34±0.01 |
|  | Moroberekan | 0.22±0.01 b | n.a. | 0.27±0.01 | 0.34±0.02 b | 0.29±0.02 b | 0.27±0.02 |
|  | 252-B | 0.30±0.01 a | n.a. | 0.27±0.02 | n.a. | 0.44±0.02 a | 0.29±0.02 |
|  | 73-B | 0.32±0.01 a | n.a. | 0.28±0.02 | n.a. | 0.43±0.01 a | 0.36±0.01 |
|  | 33-B | 0.31±0.01 a | 0.28±0.03 a | n.a. | 0.39±0.01 b | n.a. | 0.33±0.03 |
|  | 89-B | 0.31±0.01 a | 0.31±0.02 a | n.a. | 0.51±0.02 a | n.a. | 0.37±0.02 |
|  |  |  |  |  |  |  |  |
| *Genotype (p-value)* | | *< 0.001* | *< 0.01* | *0.082* | *< 0.01* | *< 0.001* | *0.059* |
|  | |  |  |  |  |  |  |
| Drought stress | Swarna | 0.01±0.01 b | 0.02±0.01 b | 0.07±0.02 b | 0 b | 0 a | 0.17±0.02 bc |
|  | Moroberekan | 0.02±0.01 b | n.a. | 0.17±0.02 a | 0.17±0.02 a | 0.17±0.03 b | 0.15±0.01 c |
|  | 252-B | 0.02±0.01 b | n.a. | 0.06±0.01 bc | n.a. | 0.02±0.01 a | 0.17±0.01 bc |
|  | 73-B | 0.03±0.02 b | n.a. | 0.02±0.01 c | n.a. | 0.02±0.01 a | 0.23±0.02 ab |
|  | 33-B | 0.12±0.02 a | 0.13±0.04 a | n.a. | 0.03±0.01 b | n.a. | 0.26±0.02 a |
|  | 89-B | 0.11±0.04 a | 0.04±0.01 b | n.a. | 0.03±0.01 b | n.a. | 0.20±0.03 abc |
|  |  |  |  |  |  |  |  |
| *Genotype (p-value)* | | *< 0.05* | *< 0.05* | *< 0.001* | *< 0.001* | *< 0.001* | *< 0.05* |
| In Experiments 13WS and 14DS QTL lines were separated into two maturity groups (E: early and L: late). Harvest index was calculated as the ratio of grain mass and total above ground mass (grain mass + shoot dry mass). Mean values ± se (n=4) are presented. Letters indicate significant difference groups within a particular treatment of each experiment. n.a.: not applicable because the genotype was not included in the experiment. | | | | | | | |

| **Table S10** Flowering time in field experiments. | | | | | | | | |
| --- | --- | --- | --- | --- | --- | --- | --- | --- |
| Treatment | Genotype | Experiment | | | | | | |
|  |  | 13DS | 13WS (E) | 13WS (L) | 14DS (E) | 14DS (L) | 14WS | 15DS |
|  |  |  |  |  |  |  |  |  |
| Well-watered | Swarna | 100 a | 102 a | 102 a | 104 a | 103±1 | 104 a | 106 a |
|  | Moroberekan | 95 b | n.a. | 91 c | 100 b | 94±5 | 84±2 e | 94±2 bc |
|  | 252-B | 94±1 c | n.a. | 91 c | n.a. | 94±2 | 93 c | 97 b |
|  | 73-B | 59 f | n.a. | 98 b | n.a. | 92 | 97±1 b | 93±1 c |
|  | 33-B | 73 e | 75 c | n.a. | 69 d | n.a. | 78 f | 80 e |
|  | 89-B | 79±1 d | 89±4 b | n.a. | 75±1 c | n.a. | 91±1 d | 86±1 d |
|  |  |  |  |  |  |  |  |  |
| *Genotype (p-value)* | | *< 0.001* | *< 0.001* | *< 0.001* | *< 0.001* | *0.61* | *< 0.001* | *< 0.001* |
|  | |  |  |  |  |  |  |  |
| Drought stress | Swarna | 142 a | 108±1 a | 110±2 a | - | - | 106 a | 120 a |
|  | Moroberekan | 100±3 b | n.a. | 93 c | 105±1 a | 98±6 | 91 cd | 98±1 b |
|  | 252-B | 105 b | n.a. | 95±1 c | n.a. | 105±5 | 95±0 bc | 99±1 b |
|  | 73-B | 100±3 b | n.a. | 102±2 b | n.a. | 99±1 | 96 b | 97 b |
|  | 33-B | 75±1 c | 82±1 c | n.a. | 67±1 c | n.a. | 83±1 e | 79±1 d |
|  | 89-B | 78±1 c | 98 b | n.a. | 79 b | n.a. | 90 d | 83 c |
|  |  |  |  |  |  |  |  |  |
| *Genotype (p-value)* | | *< 0.001* | *< 0.001* | *< 0.001* | *< 0.001* | *0.05* | *< 0.001* | *< 0.001* |
| In Experiments 13WS and 14DS, QTL lines were separated into two maturity groups (E: early and L: late). Values shown are the number  of days after sowing when 50 % of the plants in a plot had flowered. Swarna did not flower in the drought stress treatment of Experiments  14DS (E) and 14DS (L). Mean values ± se (n=4) are presented. Letters indicate significant difference groups within a particular treatment  of each experiment. n.a.: not applicable because the genotype was not included in the experiment. | | | | | | | | |

| **Table S11** Crown root number in Swarna, Moroberekan, and selected QTL lines in the field. | | | | |
| --- | --- | --- | --- | --- |
| Treatment | Genotype | Experiment | | |
|  |  | 13DS | 14DS (E) | 14DS (L) |
| Well-watered | Swarna | 394±56 | 478±42 a | 513±63 a |
|  | Moroberekan | 320±40 | 246±10 b | 238±42 b |
|  | 252-B | 375±32 | n.a. | 407±68 ab |
|  | 73-B | 422±37 | n.a. | 336±20 ab |
|  | 33-B | 387±86 | 281±38 b | n.a. |
|  | 89-B | 380±40 | 306±60 b | n.a. |
|  |  |  |  |  |
| *Genotype (p-value)* | | *0.069* | *< 0.01* | *< 0.05* |
|  | |  |  |  |
| Drought stress | Swarna | 318±69 | 413±64 a | 353±53 a |
|  | Moroberekan | 235±27 | 207±39 b | 172±11 b |
|  | 252-B | 168±20 | n.a. | 288±19 ab |
|  | 73-B | 202±30 | n.a. | 339±25 a |
|  | 33-B | 204±23 | 363±8 ab | n.a. |
|  | 89-B | 294±21 | 255±25 ab | n.a. |
|  |  |  |  |  |
| *Genotype (p-value)* | | *0.848* | *< 0.05* | *< 0.01* |
| In Experiments 13WS and 14DS QTL lines were separated into two maturity groups (E: early and L: late). Number of crown roots in BC_2_F_3_-derived lines was measured at 130, 121 and 122 days after sowing in Experiments 13DS, 14DS (E), 14DS (L), respectively. Mean values ± se (n=4) are presented. Letters indicate significant difference groups within a particular treatment of each experiment. n.a.: not applicable because the genotype was not included in the experiment. | | | | |

| **Table S12** Shoot dry mass (SDM) and total water uptake (TWU) of Swarna, Moroberekan and the QTL lines in lysimeters. | | | | |
| --- | --- | --- | --- | --- |
| Treatment | Genotype |  | SDM (g) | TWU (g) |
|  |  |  |  |  |
| Well-watered | Swarna |  | 15.5±0.9 c | 5251±520 cd |
|  | Moroberekan |  | 30.5±0.8 b | 10218±853 bc |
|  | 252-B |  | 14.8±0.9 c | 4441±849 d |
|  | 73-B |  | 16.8±0.5 c | 7706±1480 cd |
|  | 33-B |  | 21.6±2.1 bc | 15254±1821 ab |
|  | 89-B |  | 43.2±2.0 a | 16210±784 a |
|  |  |  |  |  |
| *Genotype (p-value)* | |  | *< 0.001* | *< 0.001* |
|  |  |  |  |  |
| Drought stress | Swarna |  | 12.2±1.0 ab | 3481±222 |
|  | Moroberekan |  | 13.5±0.6 ab | 3697±126 |
|  | 252-B |  | 10.9±0.3 b | 3406±113 |
|  | 73-B |  | 16.2±1.2 ab | 4050±185 |
|  | 33-B |  | 18.9±1.2 a | 4967±473 |
|  | 89-B |  | 15.2±0.3 ab | 4156±36 |
|  |  |  |  |  |
| *Genotype (p-value)* | |  | *< 0.05* | *0.255* |
| Shoot dry weight was measured at harvest (63 days after sowing; DAS) and water uptake was measured between 32 and 63 DAS. Mean values ± se (n=4-5) are presented. Letters indicate significant difference groups within a particular treatment. | | | | |

| **Table S13** Root system architecture (RSA) traits of Swarna, Moroberekan and QTL lines at seedling stage in the gel imaging platform. | | | | | | | | | | | | | | | |
| --- | --- | --- | --- | --- | --- | --- | --- | --- | --- | --- | --- | --- | --- | --- | --- |
| Genotype |  | Trait \| Condition | | | | | | | | | | | | | |
|  |  | SA (mm^2^) | |  | TL (mm) | |  | Depth (mm) | |  | Width (mm) | |  | MNR | |
|  |  | C | WD |  | C | WD |  | C | WD |  | C | WD |  | C | WD |
| Swarna |  | 1220±48 c | 1090±29 b |  | 1152±47 bc | 1109±38 a |  | 124±3 a | 118±4 a |  | 29±1 c | 26±1 b |  | 10.7±0.4 e | 8.8±0.2 d |
| Moroberekan |  | 1272±39 bc | 1066±34 b |  | 1077±33 c | 975±37 b |  | 90±2 b | 73±3 c |  | 43±2 a | 38±2 a |  | 12.1±0.5 d | 12.3±0.4 a |
| 252-B |  | 1382±35 ab | 1220±23 a |  | 1260±28 ab | 1142±40 a |  | 116±3 a | 107±5 a |  | 30±2 c | 29±1 b |  | 12.6±0.3 cd | 10.0±0.4 cd |
| 73-B |  | 1216±36 c | 1047±71 b |  | 1163±32 bc | 1071±75 ab |  | 98±5 b | 88±4 b |  | 33±2 bc | 29±1 b |  | 14.1±1.0 c | 11.7±0.3 ab |
| 33-B |  | 1388±91 ab | 888±42 c |  | 1342±101 a | 821±52 c |  | 73±7 c | 64±4 c |  | 43±2 a | 27±2 b |  | 19.9±0.8 a | 11.6±0.8 ab |
| 89-B |  | 1487±40 a | 1109±38 ab |  | 1346±36 a | 1080±34 ab |  | 95±2 b | 85±3 b |  | 40±2 ab | 36±3 a |  | 16.4±0.7 b | 11.2±0.3 bc |
|  |  |  |  |  |  |  |  |  |  |  |  |  |  |  |  |
| *Genotype*  *(p-value)* |  | *< 0.01* | *< 0.001* |  | *< 0.01* | *< 0.001* |  | *< 0.001* | *< 0.001* |  | *< 0.001* | *< 0.001* |  | *< 0.001* | *< 0.001* |
| Seedlings grown in control (C; Yoshida) and water deficit (WD; Yoshida + PEG 10%) treatments were imaged at 15 days after germination for RSA analysis.  Mean values ± se (n=26 for Swarna and Moroberekan, and n=8-12 for the QTL lines) are presented. Letters indicate significant difference groups within a particular treatment. SA: surface area; TL: total length; MNR: maximum number of roots. | | | | | | | | | | | | | | | |
