# Supplementary figures and images for: Physiological mechanisms contributing to the QTL qDTY3.2 effects on improved performance of rice Moroberekan x Swarna BC2F3:4 lines under drought
Source: Rice (N Y). 2018 Jul 31;11:43. doi: 10.1186/s12284-018-0234-1 (PMC6068063; doi:10.1186/s12284-018-0234-1)

Swarna

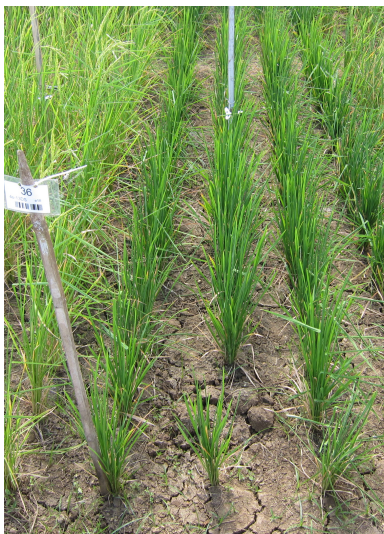

Moroberekan

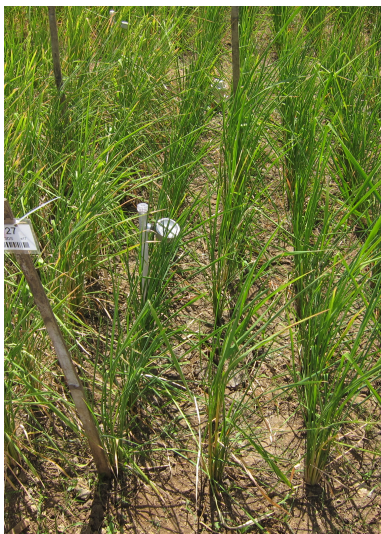

252-B

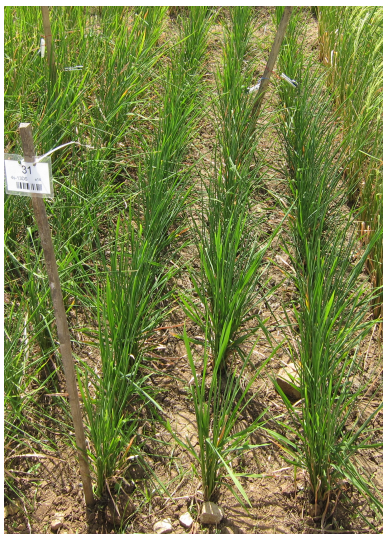

73-B

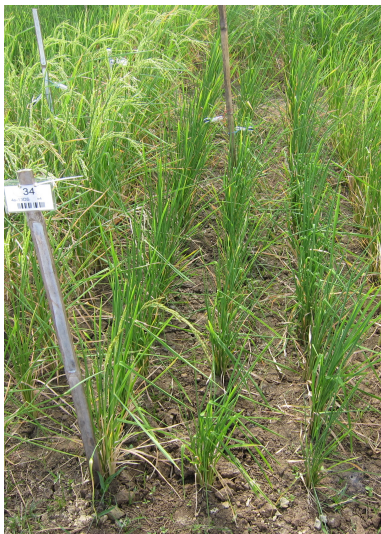

33-B

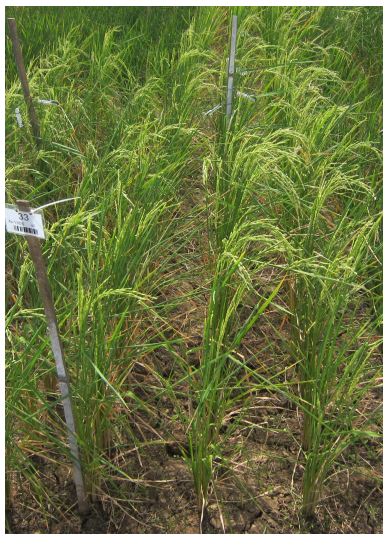

89-B

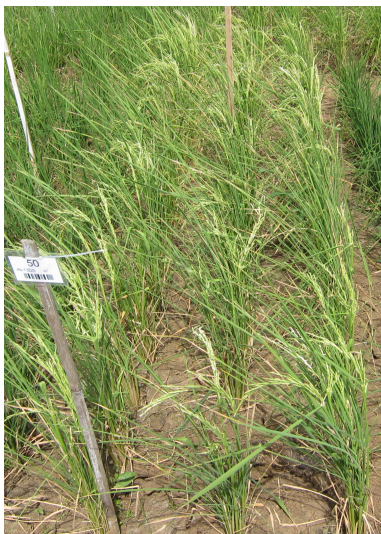

Supplement: Supplementary file 2 — Figure S1. Representative images of Swarna, Moroberekan and the four selected lines (252-B, 73-B, 33-B and 89-B) in the field Experiment 13DS. In this experiment, plots consisted of three rows spaced at 0.25 m × 15 hills spaced at 0.20 m. The first row is indicated by the labelling stick. Images were taken at 88 days after sowing that corresponded to 13 days after the imposition of the drought stress. (PDF 26584 kb) [file 12284_2018_234_MOESM2_ESM.pdf]

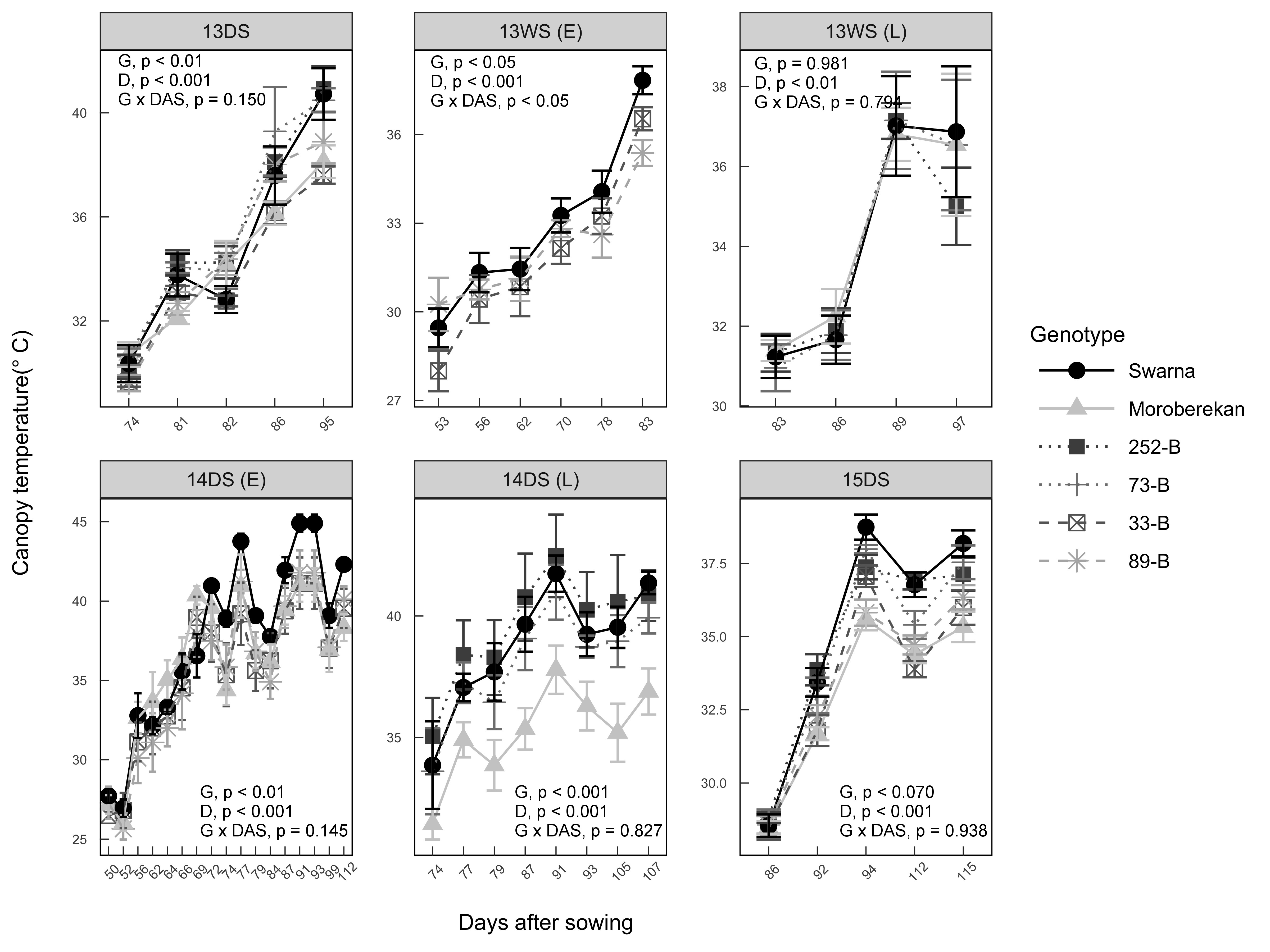

Supplement: Supplementary file 3 — Figure S2. Variation in canopy temperature of Swarna, Moroberekan, and the selected QTL lines in the drought stress treatment of Experiments 13DS, 13WS, 14DS and 15DS. In Experiments 13WS and 14DS, QTL lines were separated into two maturity groups (E: early and L: late). Mean values ± se (n = 4) are presented and p-values shown are for genotypic (G), dates (days after sowing: DAS) and genotype × date (G × DAS) differences for canopy temperature calculated across the different dates. (JPEG 1015 kb) [file 12284_2018_234_MOESM3_ESM.jpeg]

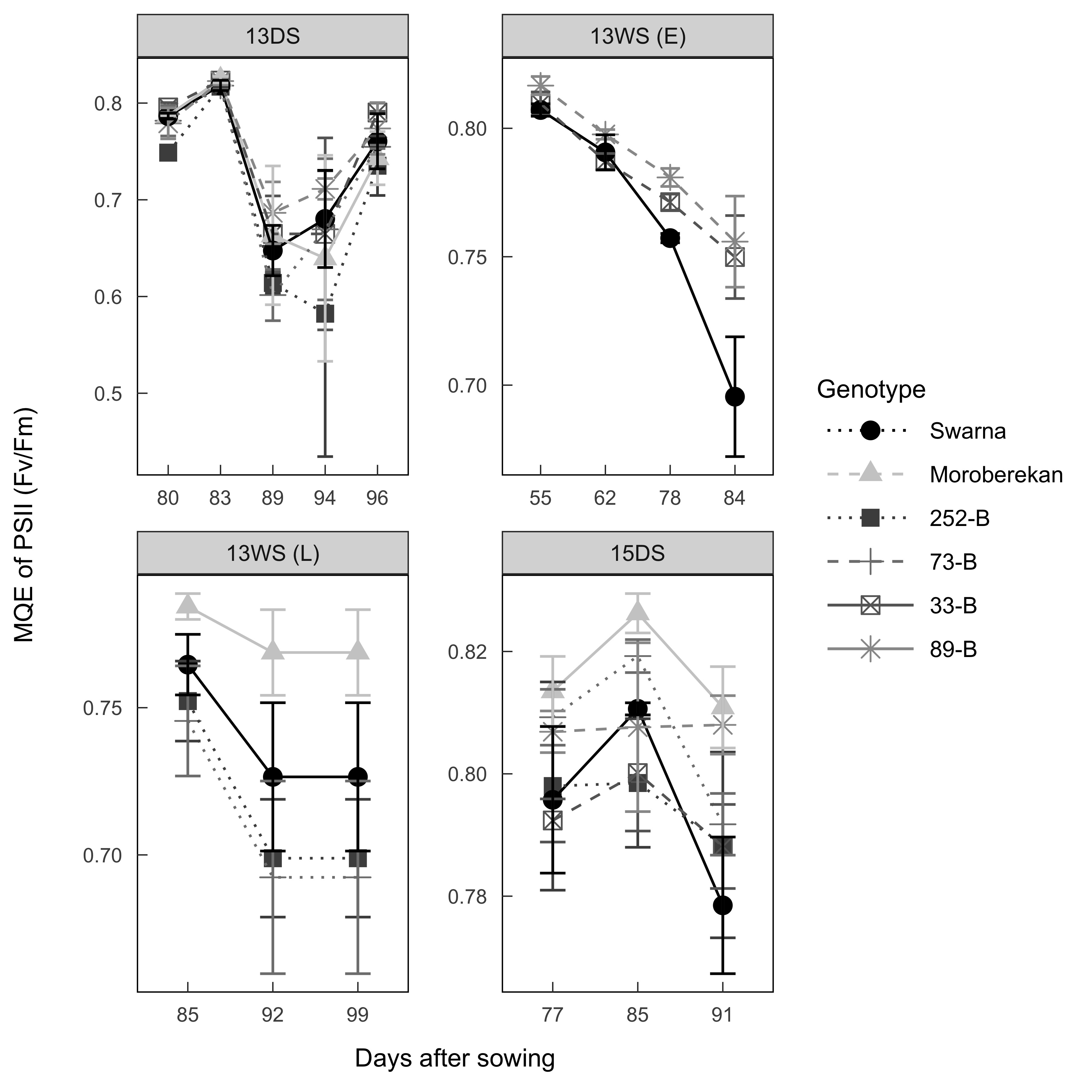

Supplement: Supplementary file 4 — Figure S3. Variation in maximum quantum efficiency (MQE, Fv/Fm) of photosystem II (PSII) in Swarna, Moroberekan, and the selected QTL lines in the drought stress treatment of field experiments. Fv/Fm was typically measured between 9 AM to 11 PM at sunny and not windy times using a Handy Pea chlorophyll fluorometer (Hansatech Instruments Ltd., England) at different dates during soil dry-down. Two fully expanded leaves from 2 different plants were used per plot (flag leaves were excluded). The leaf area subjected to the measurement was dark-adapted for at least 30 min using leaf clips of 4 mm diameter firmly attached to bamboo sticks. In Experiment 13WS, QTL lines were separated into two maturity groups (E: early and L: late). Mean values ± se (n = 4) are presented. (JPEG 658 kb) [file 12284_2018_234_MOESM4_ESM.jpeg]

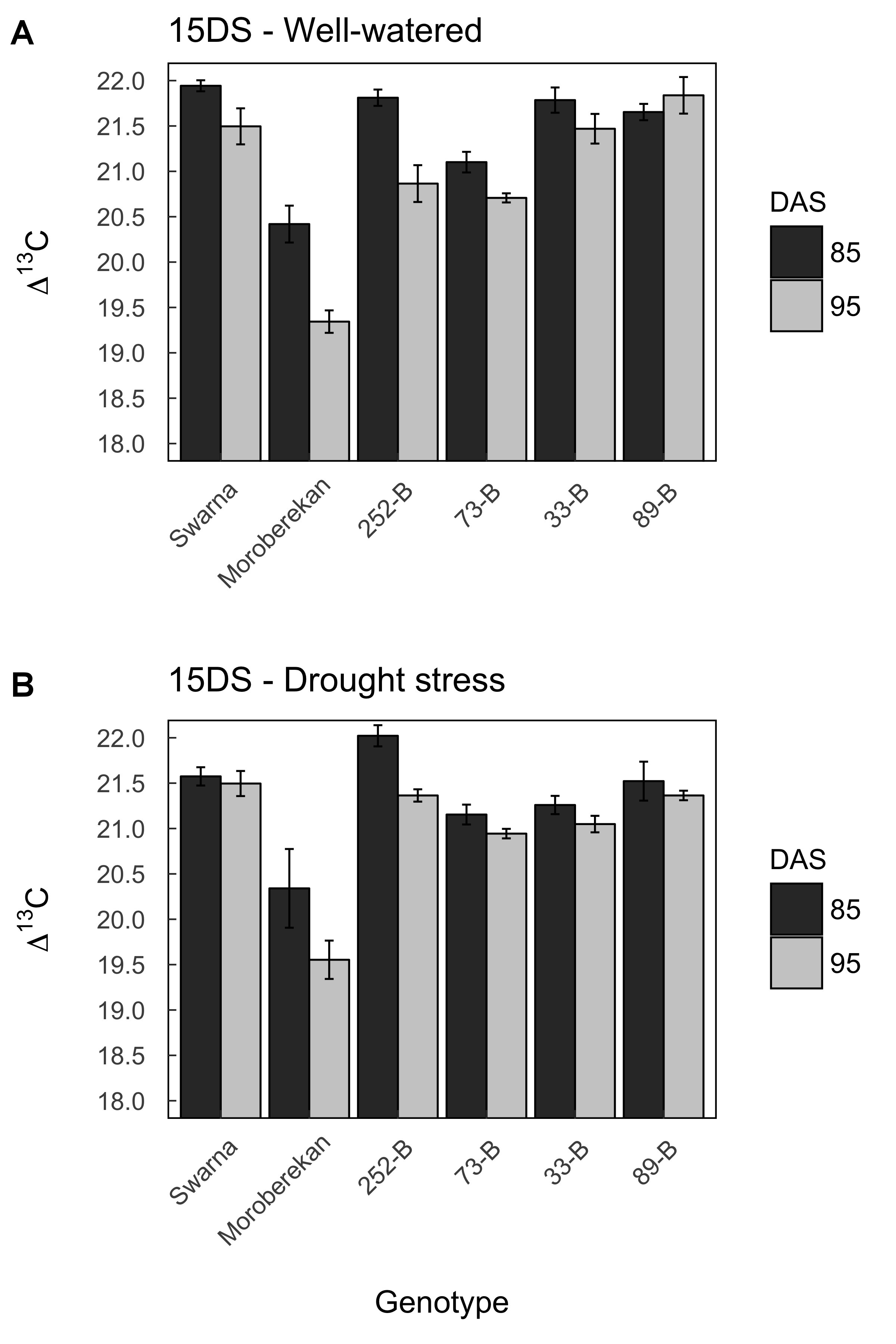

Supplement: Supplementary file 5 — Figure S4. Carbon isotope discrimination (Δ13C) in Swarna, Moroberekan and the selected QTL lines in Experiment 15DS. Δ13C was measured on the youngest fully-formed leaves collected at 85 and 95 DAS (days after sowing) in both well-watered and drought stress treatments of Experiment 15DS using the following formula: (− 8 – leaf 13C concentration) / [1 + (leaf 13C concentration/1000)] (Farquhar et al. 1989). Bars show mean ± se (n = 4). (JPEG 401 kb) [file 12284_2018_234_MOESM5_ESM.jpeg]

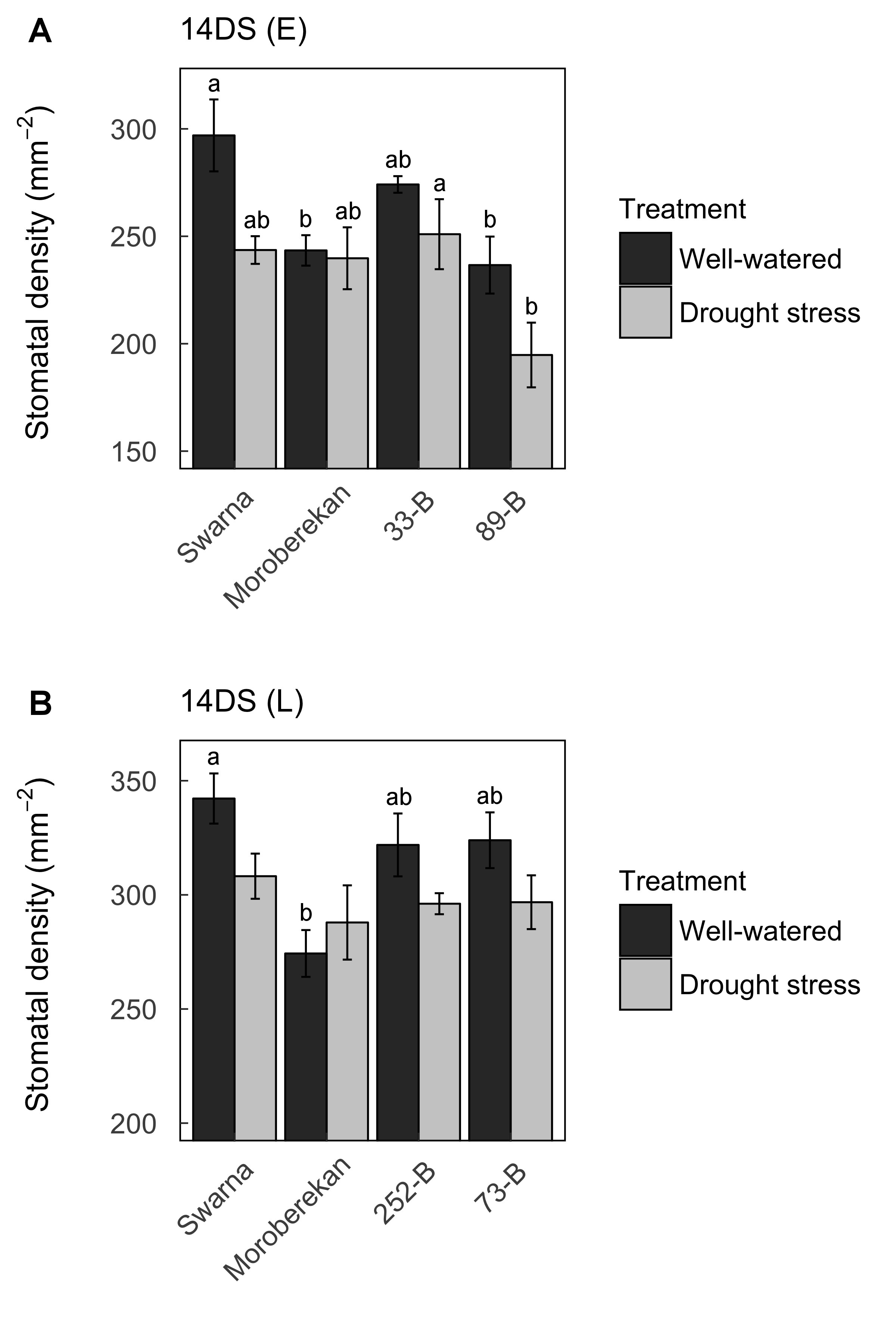

Supplement: Supplementary file 6 — Figure S5. Stomatal density of Swarna, Moroberekan, and QTL lines in the field. Stomatal density was measured during the reproductive stage following the procedure of Kusumi et al. (2012). Epidermal imprints of the adaxial surface of three fully expanded leaves per plot were collected and the stomata present in an area of about 0.3 mm2 were counted under a microscope at 100× magnification. In Experiment 14DS, QTL lines were separated into two maturity groups (E: early and L: late). Bars show mean ± se (n = 4) and letters indicate significant difference groups within treatments. (JPEG 376 kb) [file 12284_2018_234_MOESM6_ESM.jpeg]

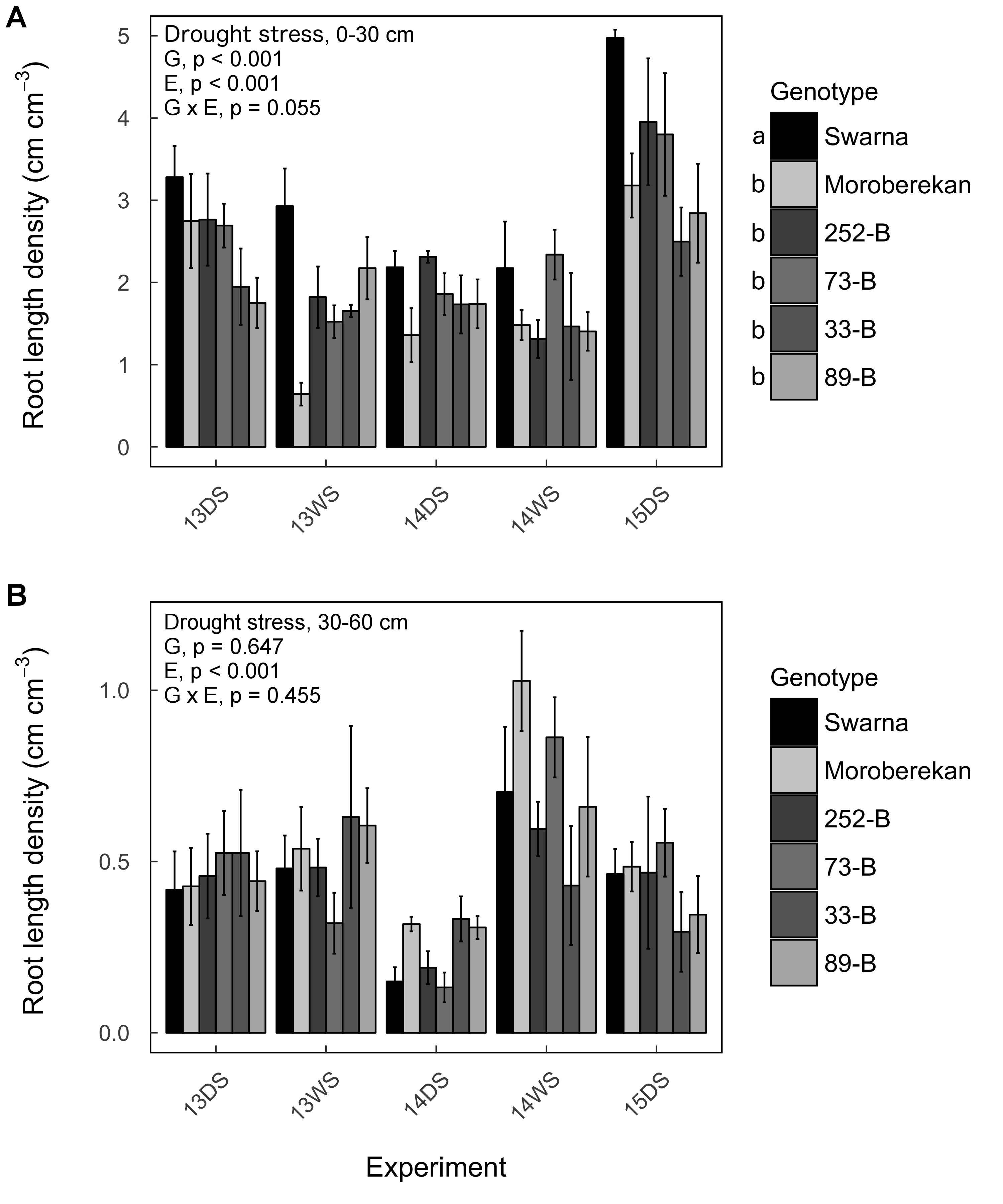

Supplement: Supplementary file 7 — Figure S6. Root length density (RLD) of Swarna, Moroberekan and the four selected QTL lines under drought stress conditions in the field. Bars represent mean root length density (n = 3–4) from 0 to 30 cm (A) and 30–60 cm (B) of each experiment. Results of RLD for Swarna and Moroberekan grown in the late maturity group of Experiments 13WS and 14DS are presented. P-values shown are for genotypic (G), experimental (E) and genotype × experiment (G × E) differences for RLD calculated across the different experiments. Letters indicate different significance groups. (JPEG 574 kb) [file 12284_2018_234_MOESM7_ESM.jpeg]

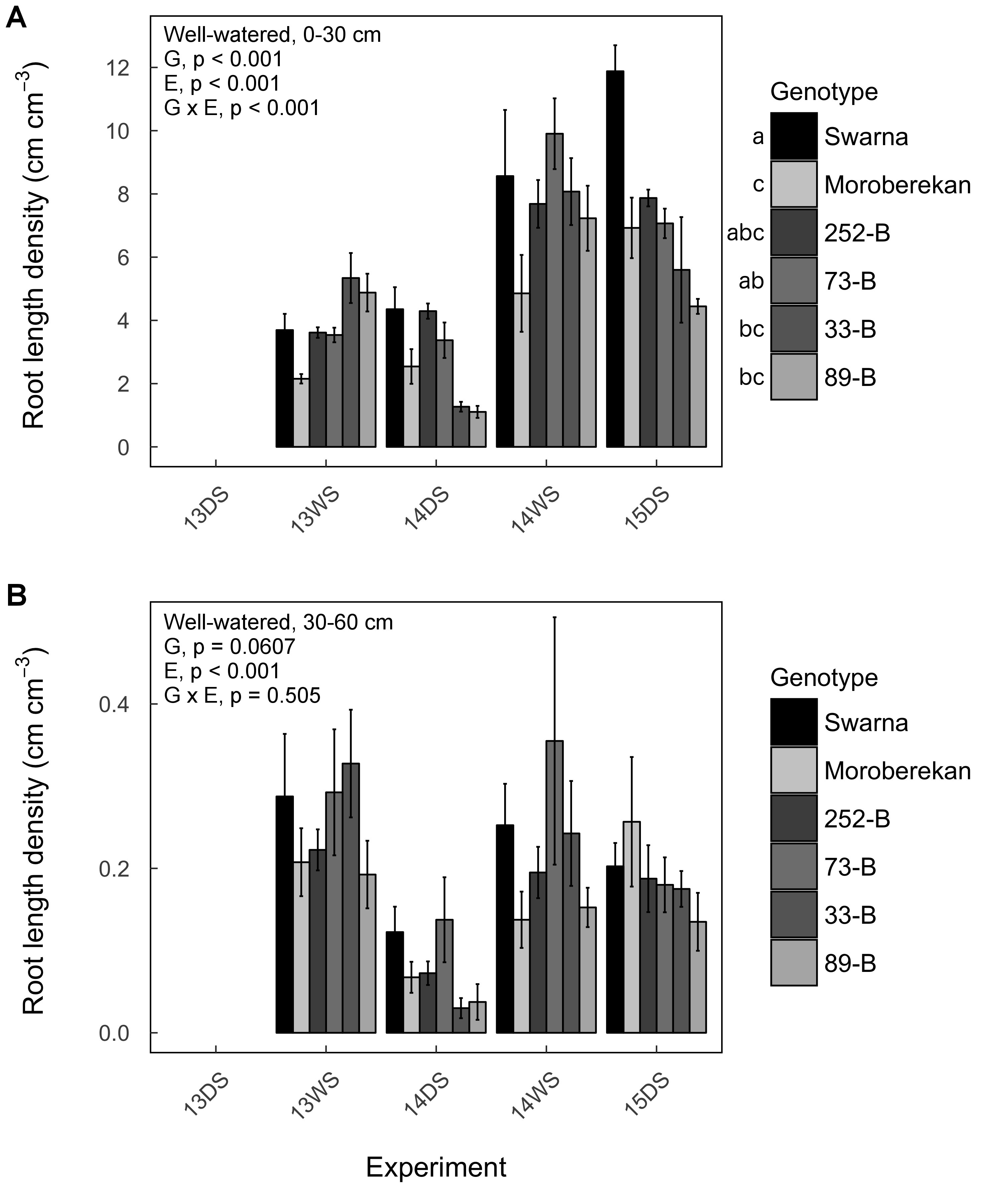

Supplement: Supplementary file 8 — Figure S7. Root length density (RLD) of Swarna, Moroberekan and the four selected QTL lines under well-watered conditions in the field. Bars represent mean root length density (n = 4) from 0 to 30 cm (A) and 30–60 cm (B) of each experiment. RLD was not measured in the well-watered treatment of Experiment 13DS. Results of RLD for Swarna and Moroberekan grown in the late maturity group of Experiments 13WS and 14DS are presented. P-values shown are for genotypic (G), experimental (E) and genotype × experiment (G × E) differences for RLD calculated across the different experiments. Letters indicate different significance groups. (JPEG 541 kb) [file 12284_2018_234_MOESM8_ESM.jpeg]

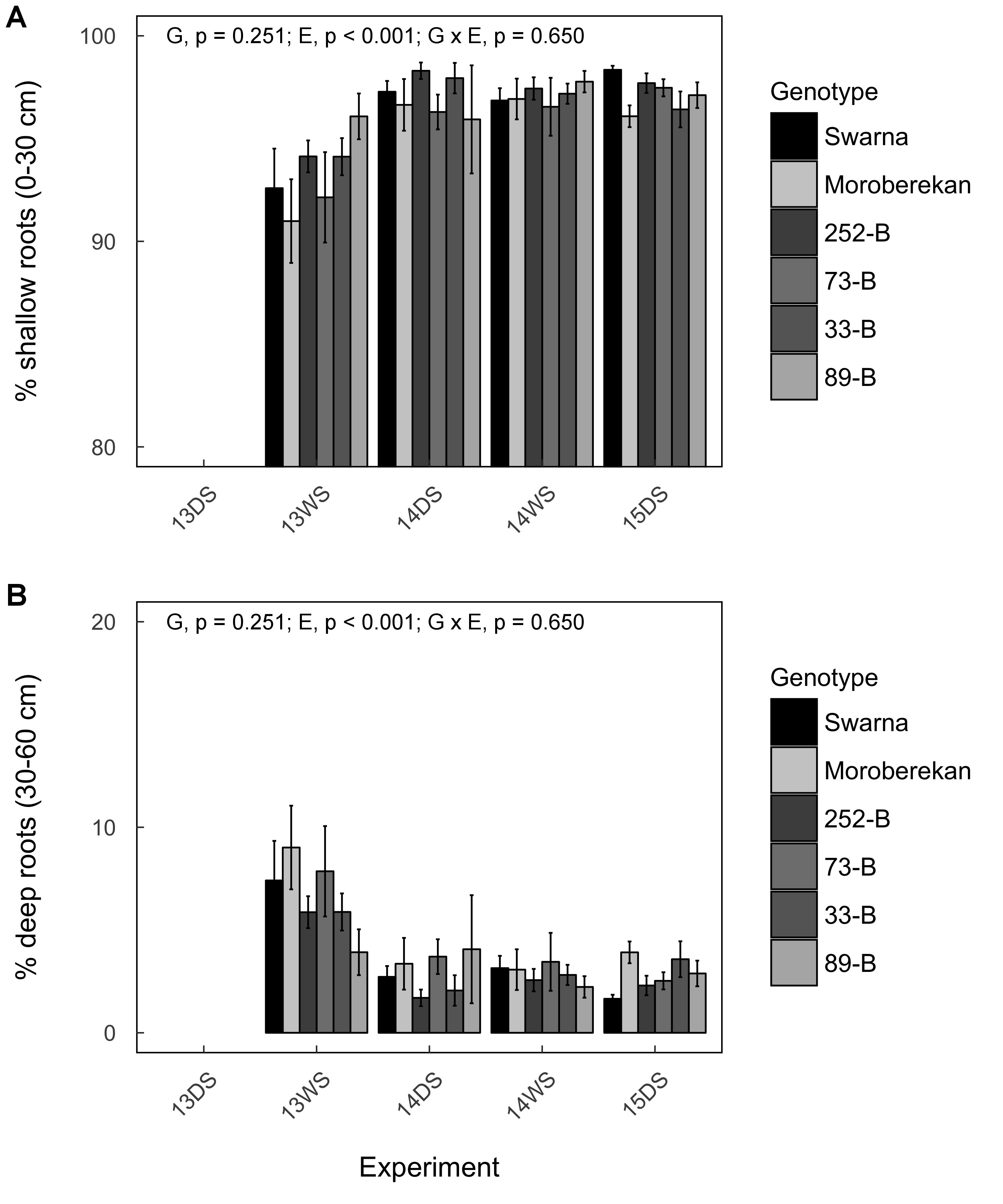

Supplement: Supplementary file 9 — Figure S8. Percentage of shallow and deep root length in Swarna, Moroberekan and the four selected QTL lines under well-watered conditions in the field. Total root length measured in 15-cm soil segments from 0 to 60 cm were analyzed to calculate percent (%) shallow (from 0 to 30 cm; A) and deep (from 30 to 60 cm; B) roots. Total root length was not measured in the well-watered treatment of Experiment 13DS. Results from the late maturity group of Experiments 13WS and 14DS are presented for Swarna and Moroberekan. Bars represent mean values ± se (n = 4). P-values shown are for genotypic (G), experimental (E) and genotype × experiment (G × E) differences calculated across the different experiments. (JPEG 511 kb) [file 12284_2018_234_MOESM9_ESM.jpeg]

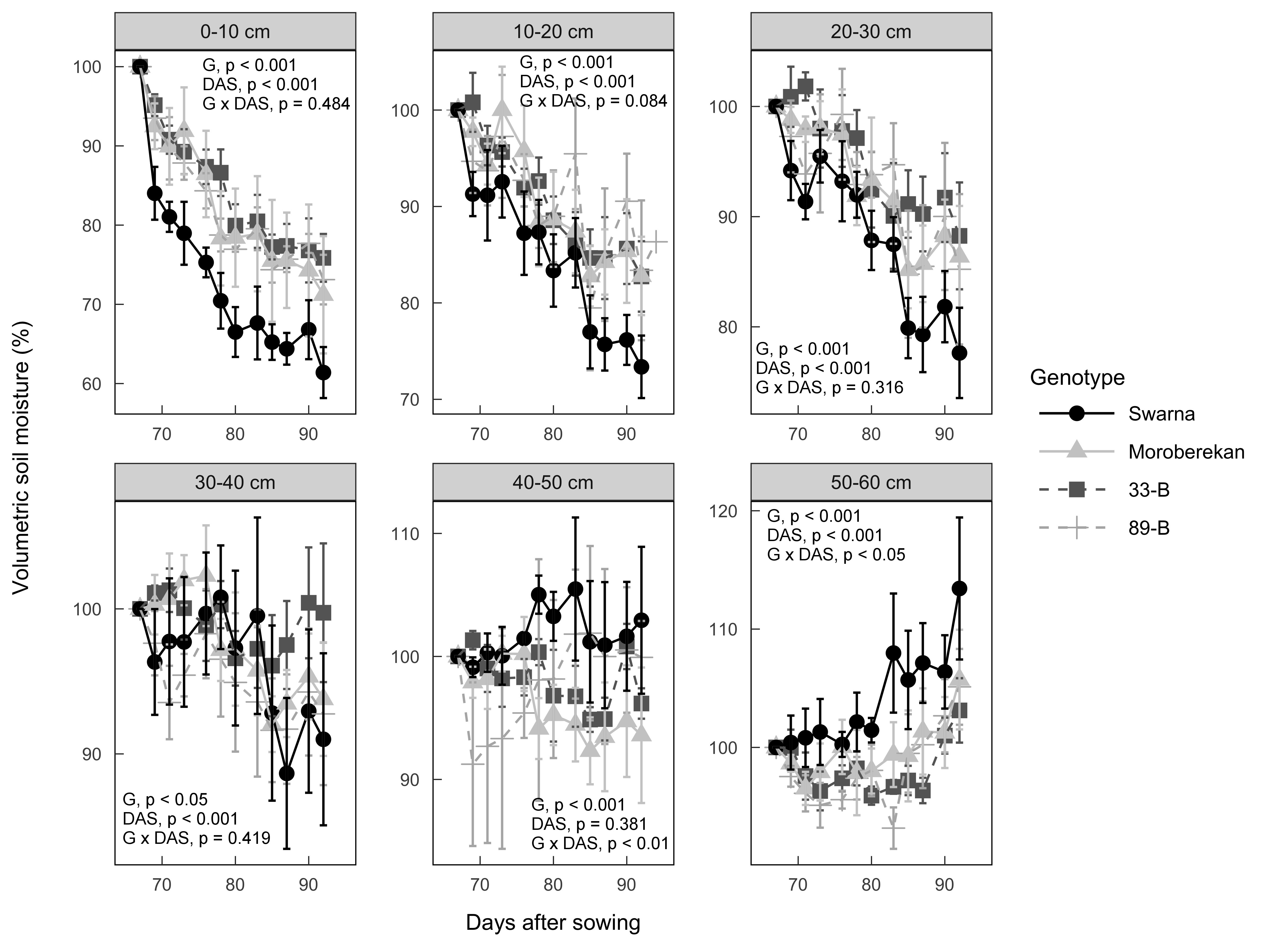

Supplement: Supplementary file 10 — Figure S9. Variations of volumetric soil moisture in the drought-stressed plots of Swarna, Moroberekan, 33-B, 89-B in Experiment 14DS (E). Soil moisture was expressed as percent of initial soil moisture after initiation of the drought stress at 60 days after sowing (DAS). Mean values ± se (n = 4) are presented and p-values shown are for genotypic (G), dates (DAS) and genotype × date (G × DAS) differences for volumetric soil moisture calculated across the different dates. (JPEG 1034 kb) [file 12284_2018_234_MOESM10_ESM.jpeg]

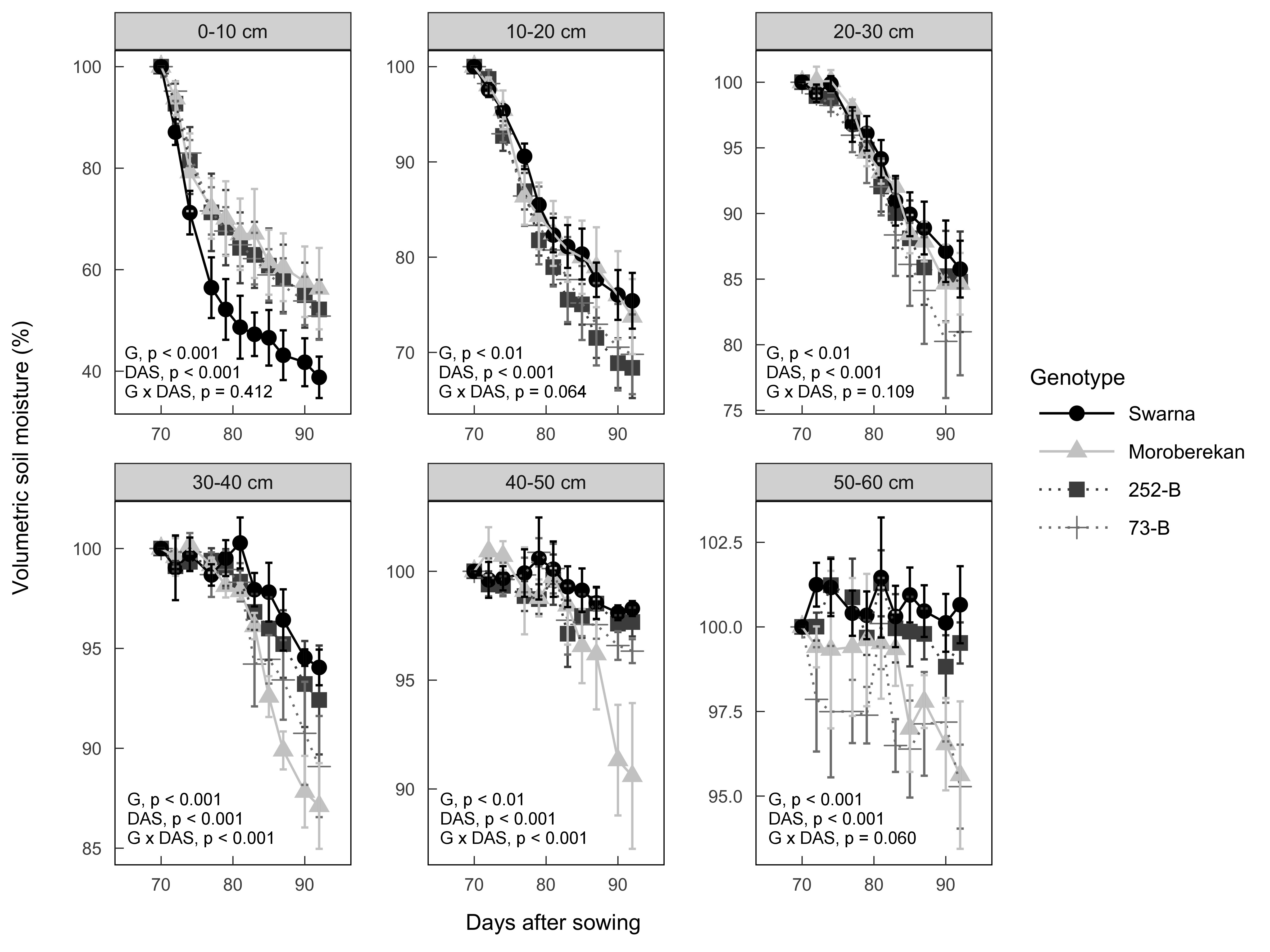

Supplement: Supplementary file 11 — Figure S10. Variations of volumetric soil moisture in the drought-stressed plots of Swarna, Moroberekan, 252-B and 73-B in Experiment 14DS (L). Soil moisture was expressed as percent of initial soil moisture after initiation of the drought stress at 70 days after sowing (DAS). Mean values ± se (n = 4) are presented and p-values shown are for genotypic (G), dates (DAS) and genotype × date (G × DAS) differences for volumetric soil moisture calculated across the different dates. (JPEG 941 kb) [file 12284_2018_234_MOESM11_ESM.jpeg]

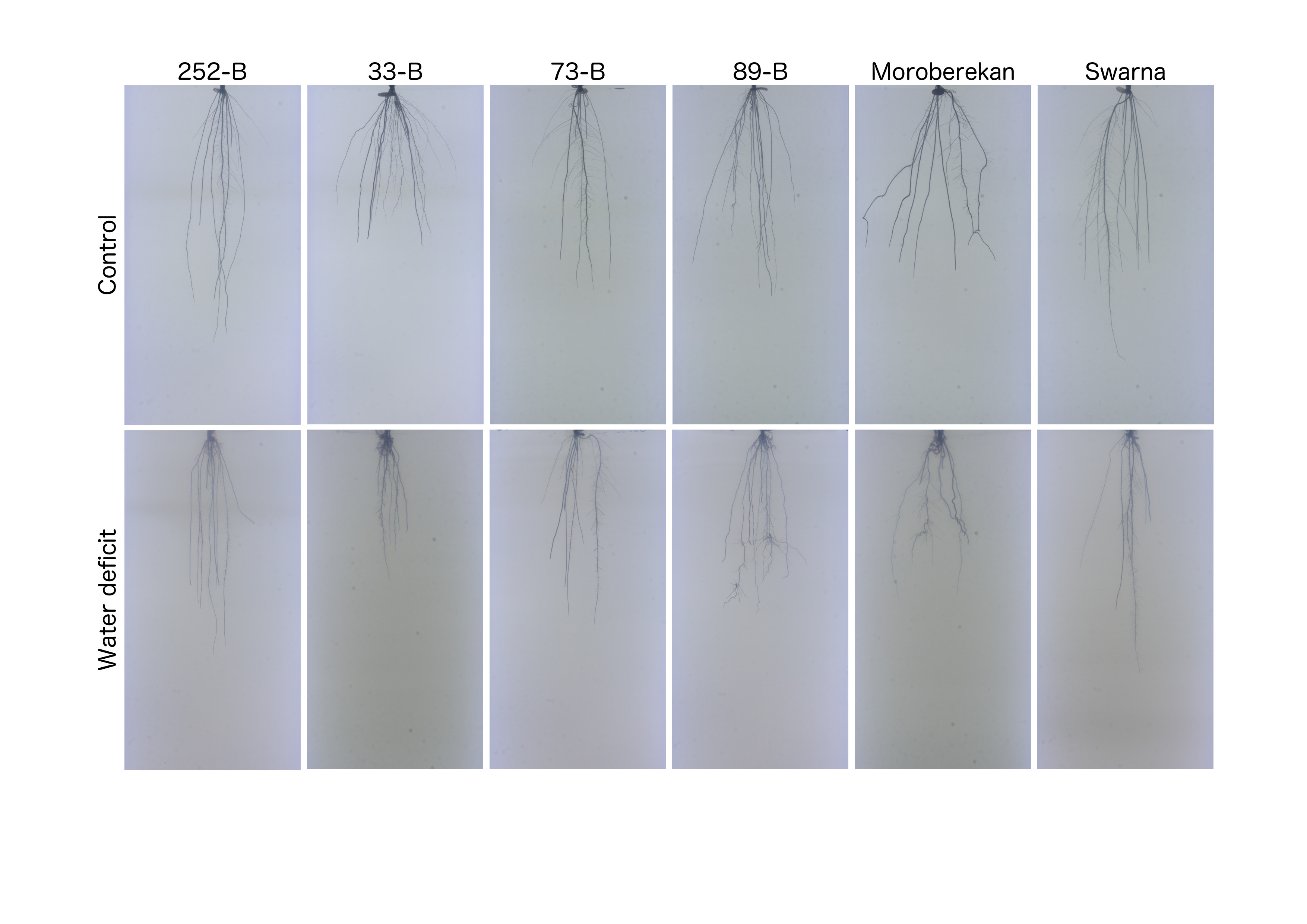

Supplement: Supplementary file 12 — Figure S11. Representative images of the root system of Swarna, Moroberekan, and the QTL lines grown in the gel imaging platform. Seedlings grown under control (Yoshida) or water deficit (Yoshida + PEG 10%) conditions were imaged at 15 days after germination or 12 days after transplanting. (JPEG 600 kb) [file 12284_2018_234_MOESM12_ESM.jpeg]
